# Addressing unpredictability may be the key to improving performance with current clinically prescribed myoelectric prostheses

**DOI:** 10.1038/s41598-021-82764-6

**Published:** 2021-02-08

**Authors:** A. Chadwell, L. Kenney, S. Thies, J. Head, A. Galpin, R. Baker

**Affiliations:** 1 Centre for Health Sciences Research, University of Salford, Salford, UK; 2 Salford Business School, University of Salford, Salford, UK

**Keywords:** Trauma, Health care, Medical research

## Abstract

The efferent control chain for an upper-limb myoelectric prosthesis can be separated into 3 key areas: signal generation, signal acquisition, and device response. Data were collected from twenty trans-radial myoelectric prosthesis users using their own clinically prescribed devices, to establish the relative impact of these potential control factors on user performance (user functionality and everyday prosthesis usage). By identifying the key factor(s), we can guide future developments to ensure clinical impact. Skill in generating muscle signals was assessed via reaction times and signal tracking. To assess the predictability of signal acquisition, we inspected reaction time spread and undesired hand activations. As a measure of device response, we recorded the electromechanical delay between electrode stimulation and the onset of hand movement. Results suggest abstract measures of skill in controlling muscle signals are poorly correlated with performance. Undesired activations of the hand or incorrect responses were correlated with almost all kinematics and gaze measures suggesting unpredictability is a key factor. Significant correlations were also found between several measures of performance and the electromechanical delay; however, unexpectedly, longer electromechanical delays correlated with better performance. Future research should focus on exploring causes of unpredictability, their relative impacts on performance and interventions to address this.

## Introduction

Myoelectric prostheses are designed to provide aesthetic appearance and a degree of upper-limb functionality for people with upper-limb absence. Self-reported rejection of these prostheses is high, with control of the prosthesis being commonly cited as one of the primary reasons^1–4^. This observation may indicate that the significant engineering efforts aimed at improving prosthesis control^5^ may not have been addressing the most important issues. Aside from the study by Atkins^1^, qualitative studies often fail to clearly define what is meant by terms such as control and functionality. It is possible therefore, that participants in such studies are referring to different factors, when reporting ‘control problems’ as one of the primary reasons for rejection.

The efferent prosthesis control chain can be broken down into three key areas:

1. Signal generation—If a person is unable to generate the required electromyographic (EMG) signals then it would be anticipated that they would struggle to gain fine control over the prosthetic hand. Studies have shown that it is possible to learn to control the level of activation of EMG signals to a high level of speed and accuracy^6–8^. However, there is also ambiguity around whether improvements in EMG control assessed using abstract on-screen tasks, transfer to improved control over the prosthetic hand itself^9–13^.
2. Signal acquisition—Regardless of the quality of signal generation, if the interface between the electrodes and the skin does not allow for accurate and reliable signal transduction, then the user will have trouble controlling the device. If the socket is too loose, the arm will move around within the socket and the electrodes may lose contact with the skin leading to signal artefacts, or an inability to activate the hand. A clear relationship between the tightness of the electrodes and unwanted prehensor activation has been previously shown^14^, and it is not uncommon for users to report their device activating unexpectedly, becoming stuck in a closed position, or simply breaking down^15^. Additional loads carried within the hand may exacerbate these artefacts.
3. Device response—The addition of a delay to a perfectly predictable system can make control difficult. The central nervous system must work with old information and consequently it is more difficult to control the prosthesis and adapt to perturbations. Hence, delays in a system in which there is already inherent unpredictability, are likely to make control very challenging indeed. Farrell^16^ indicated that the optimal prosthesis controller delay for 90% of the population, allowing sufficient time for signal analysis, is 100–125 ms, whilst the actual electromechanical delay in clinically prescribed prostheses is currently unpublished.

In order to understand which improvements to the control chain would have the greatest impact on improving user performance, we investigated the relationships between each of these control factors and, two aspects of performance: (1) the prosthesis user’s ability to use their prosthesis, termed their functionality (assessed within the lab/clinic using measures of task success, duration, kinematics, and gaze behaviours) and (2) how they actually use the prosthesis, termed everyday prosthesis usage (assessed in the real-world using measures of prosthesis wear time and symmetry of upper-limb activity).

When we began our work, we asked the following questions:

1. Are measures of functionality, assessed using a multi-stage goal-directed task within a lab-based/clinical environment, correlated with measures of everyday prosthesis usage?
2. Are measures relating to aspects of the prosthesis control chain associated with measures of user performance (functionality and everyday prosthesis usage)?

In a previous paper^17^ we reported the findings from question 1. In summary, we found no correlations between any of our measures of functionality and the measures of everyday prosthesis usage. This suggests that the degree to which a user is able to control their prosthesis may have limited influence on the multiple and context-specific decisions on wear and/or use of their prosthesis in everyday life. Further we highlighted the absence of a correlation between prosthesis wear and prosthesis use, underlining the importance of differentiating these two measures.

In this new article we address question 2, with the aim of informing the key areas for future research and development efforts.

## Methods

Each participant attended a 3–4 h testing session, and was provided with breaks throughout including a half hour break mid-way.

The methodology for the measurement of signal generation (hereafter referred to as ‘EMG skill’) and signal acquisition (hereafter referred to as ‘unpredictability’) have previously been published in full alongside details on the assessment of functionality using our multi-stage ‘cylinder task’^18^. The methods for the assessment of the user’s everyday prosthesis usage have also been published in full^19^. In this article we will therefore only summarise these methods and refer where necessary to the methodology papers for further details. The methods for the assessment of prosthesis response (hereafter referred to as the ‘electromechanical delay’) have not previously been published, therefore these will be covered in full within this article. Further details on the design of customised setups (such as the reaction time box) can be found in the appendices to Chadwell’s PhD thesis^20^.

### Ethical approval

Ethical approval for this study was granted by the University of Salford School of Health Sciences Research Ethics committee (REF: HSCR 16-25), by the University of Strathclyde Department of Biomedical Engineering Ethics Committee (DEC.BioMed.2017.220) and through the NHS IRAS system (IRAS Project ID: 193794). Informed consent was gained from all participants. The study abided by the ethical principles underlying the Declaration of Helsinki and good practice guidelines on the proper conduct of research. Regulations relating to the protection of subject data were followed including section 251 of the NHS Act 2006 and the Data Protection Act 1998.

### Participants

Participants with unilateral upper-limb absence at a trans-radial level were included in this study. All participants had been prescribed a single degree of freedom myoelectric prosthesis (e.g. Steeper Select or Ottobock DMC Plus/VariPlus/Sensor Speed). Regular use of the prosthesis was not a requirement.

Twenty participants (14 male, 6 female), age range 18–75 (mean age 53) were recruited. Time since prescription of a myoelectric prosthesis ranged from 1.5 to 39 years (mean 20). Eleven people were recruited with congenital limb absence (6 Right/5 Left), and nine with an amputation (6 Right/3 Left); six of the amputations had occurred on the dominant side. Time since amputation ranged from 8 to 47 years (mean 25).

Four NHS mobility centres were involved in this study. Seven participants were recruited from Manchester Specialised Ability Centre, four from the Douglas Bader Rehabilitation Centre in Roehampton, three from Sheffield Mobility and Specialised Rehabilitation Centre, and two from the Nottingham Mobility Centre. An additional two participants were recruited through their links with the University of Salford, and two through their links with the University of Strathclyde.

### Protocol for the assessment of control factors

#### EMG skill

**Figure 1:**
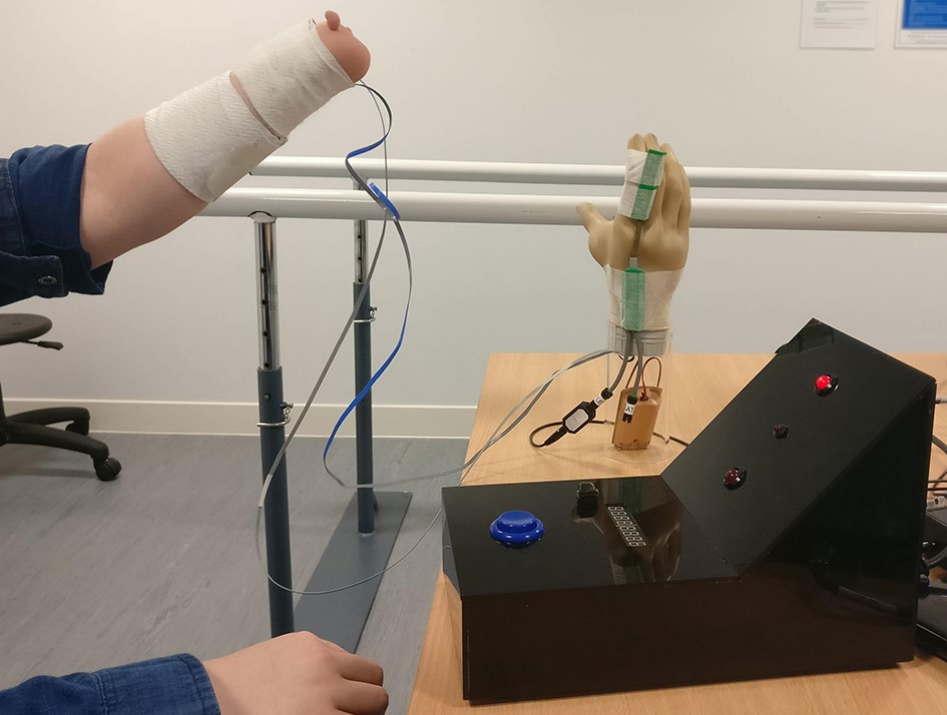
“Ideal” electrode skin interface. The electrode positions and gain settings were determined as described in Chadwell et al.^18^. These involve a mapping process starting at the muscle bulk and ending at the position where the signal is maximised, followed by adjustment of the electrode gain visualised using the Myoboy system developed by Ottobock Gmbh. To avoid movement of the electrodes, an elasticated bandage is used to hold them in place. The prosthetic hand is placed on the tabletop. Please note that this image shows a participant completing part of the unpredictability assessment. For the skill assessment, the forearm was held horizontally.

Three separate tasks were used to assess the EMG skill of the user, these will be referred to as: (1) intuition task, (2) static tracking task, and (3) dynamic tracking task. For all these tasks, the “ideal” electrode placement introduced in Chadwell et al.^18^ was used (Fig. 1). This involved holding the electrodes tightly against the skin of the residual limb using an elastic bandage whilst the prosthetic hand was placed on the tabletop; the optimal position for the electrodes and the gain settings were determined using the methods taught to student prosthetists at the University of Salford (see Chadwell et al. for details^18^).

The intuition task aimed to measure the time taken for the user to decide which muscles to contract in order to open (wrist extensors) or close (wrist flexors) the prosthetic hand. This task consisted of simple and choice reaction time tasks (SRT & CRT tasks)^18^. Each trial began with the prosthetic hand in a neutral aperture position. A custom-made reaction time box was produced (Fig. 1) with two 10 mm red LEDs to act as stimuli (Top = Open, Bottom = close) and a 5 mm central LED to focus the participant’s attention at the start of each trial. The onset of prosthetic hand movement was detected using an electronic goniometer (Biometrics Ltd. Sampling rate 1000 Hz) placed across the proximal joint of the index finger (details of onset detection are provided as supplementary material to Chadwell et al.^18^ and in the appendices to Chadwell’s thesis^20^). For the simple task participants were informed in advance as to the response to make to the stimulus, whereas with the choice task two separate stimuli were used and the specific stimuli informed the participant as to whether they should generate an open or close signal in response. Participants completed 10 trials of the SRT hand opening task, followed by 10 trials of the SRT hand closing task. 20 CRT trials were then undertaken. Trials where the participant reacted early (before 100ms^21^), reacted late (after 1000ms^21^), or responded with the incorrect response (e.g. opened the hand instead of closing) were excluded from the analysis. The participant’s decision time was calculated based on the difference between the mean CRT and mean SRT. A long decision time suggests that the participant found the decision as to which muscle they needed to contract less intuitive.

The tracking tasks aimed to establish the level of control the user had over the amplitude of the EMG signals. Both tracking tasks used a Myoboy system with PAULA software developed by Ottobock Gmbh. This is an off-the-shelf product available in several prosthetic clinics for the prescription and training of myoelectric prostheses.

The static tracking task^18^ used the PAULA signal visualisation screen and involved the participant sustaining their EMG signal amplitude between on screen boundaries of 39 and 51 for a period of 3 s (Note that the units for these post-processed signal values are not provided by Ottobock). Participants were scored based on the percentage of time the signal stayed within the boundaries. The boundary levels were chosen following pilot testing to generate a wide spread of scores^18^. Three attempts were given for each muscle (open then close) for the participant to get the best scores they could.

The dynamic tracking task^18^ used a “car game” integrated into the PAULA software. The game involved the participant steering a car past on-screen obstacles which moved from the right side of the screen to the left; the height of the car on the screen was controlled using the amplitude of the EMG signal. Participants were scored based on the percentage of obstacles avoided (best of 2 trials). Initially participants were given one car to control, first using their open signal (2 trials) and then using their close signal (2 trials). Finally, participants were given two cars to control simultaneously (one car for each muscle); again, two trials were performed by the participants and the highest score was selected. The obstacles were presented with the first requiring a contraction of the muscle and the next requiring relaxation. For the two-car task one car followed one obstacle behind the other so that when one muscle was contracted, the other muscle should remain relaxed.

Full details of all these assessments with additional figures are provided in Chadwell et al.^18^.

#### Unpredictability

To understand the unpredictability introduced at the interface between the electrodes and the skin, three different conditions were evaluated.

**Figure 2:**
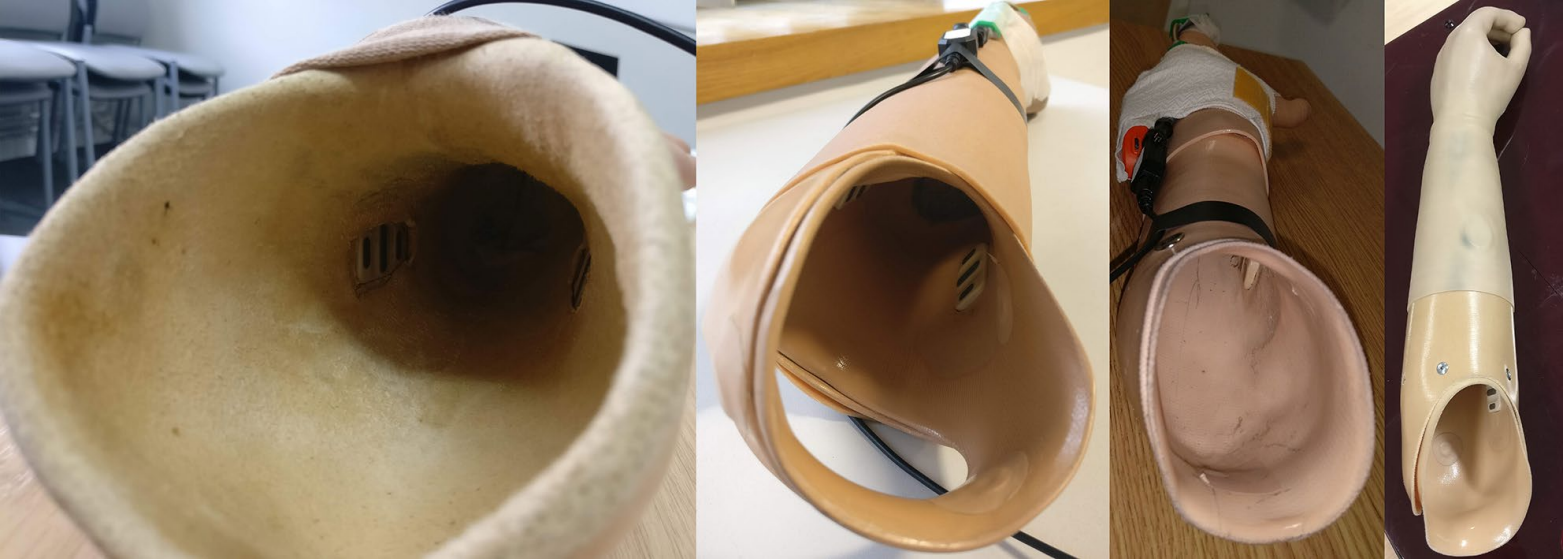
Socket mounted electrodes. Images from participants’ own clinically prescribed prostheses. Note that in the far-left image, the quality of the skin–electrode interface is disrupted by a leather shim on the interior of the socket.

Two of these conditions explored participants’ performance when the user’s own prosthesis was worn, and hence the electrodes were socket-mounted (Fig. 2). As the ability to reliably and quickly generate EMG signals in response to prompts will vary across participants, task performance was also assessed using the “ideal” skin–electrode interface (Fig. 1) to act as a baseline level of unpredictability.

Condition 1 (baseline): The “ideal” skin–electrode interface (described in ‘EMG skill’ above) involved holding the electrodes against the arm using an elasticated bandage to avoid unwanted movement against the skin.

Condition 2 (own prosthesis): The first socket condition involved the participant wearing their own prosthetic socket (with embedded electrodes).

**Figure 3:**
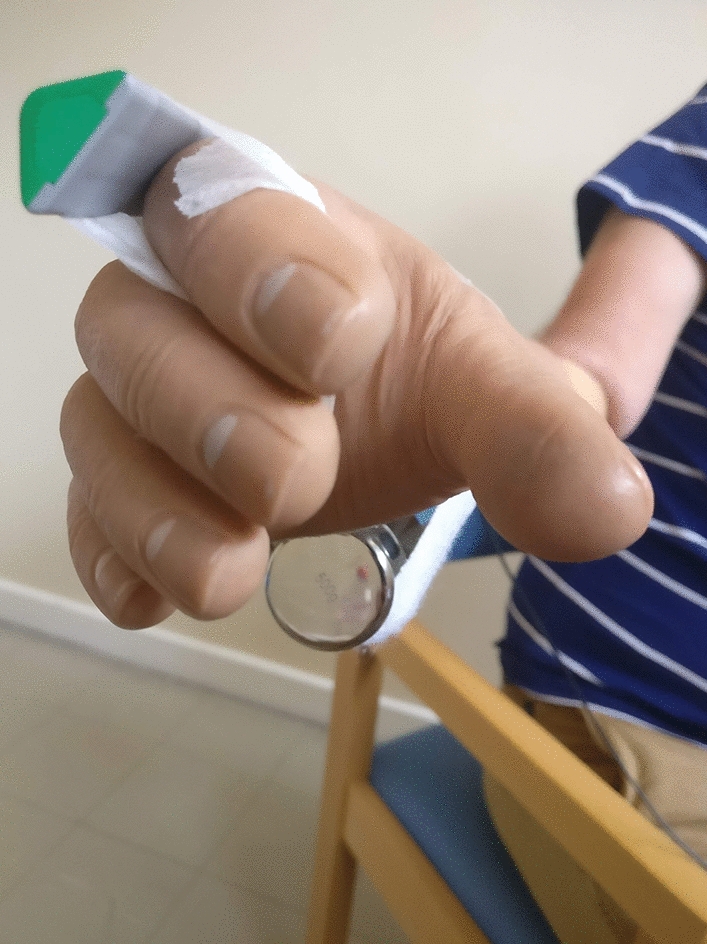
500 g Load added to prosthetic hand. The load was bandaged to the hand at the proximal palm/distal wrist level so as not to disrupt the opening/closing of the hand.

Condition 3 (own prosthesis + load): The second socket condition included the addition of a 500 g load to the hand to simulate the effects of carrying an object (Fig. 3).

For all analyses, the two socket conditions (conditions 2 and 3) were combined.

Two aspects of unpredictability were assessed: (1) whether the hand responded as desired when the participant attempted to activate it, and (2) whether there was undesired activation of the hand when the participant did not attempt to activate it.

The desired activation was evaluated using simple reaction time tasks^18^. More variable reaction times suggest higher unpredictability in the prosthesis response. To exacerbate the problems with unpredictability experienced by some users, the arm was placed in two arm orientations known to interfere with the contact between the socket mounted electrodes and the skin (45° above and below the horizontal) (see Chadwell et al.^18^ and Fig. 1 for more details). Participants completed 20 open trials and 20 close trials for the “ideal” interface. For each of the socket conditions 10 open and 10 close trials were undertaken. The spread in the reaction times and the number of occasions where the hand did not respond or responded incorrectly were recorded. Unsuccessful trials were identified as those where the participant responded early (< 100 ms) or late (> 1000 ms)^21^ and those where the participants moved the hand in the wrong direction (i.e. opening during a closing trial). The difference in the spread of reaction times between the socket conditions and the baseline “ideal” interface condition was taken as a measure of the unpredictability in the desired response caused by the prosthetic socket (Standard Deviation__Socket_ – Standard Deviation__Ideal_). A positive difference would suggest that the response of the prosthetic hand was less predictable with the socket housed electrodes than when using an “ideal” interface.

To assess the number of undesired activations, participants were asked to move their arm between the two positions (± 45°), while not activating the hand. Each transition began with the hand either fully open or fully closed. The number of undesired activations of the hand were recorded (24 transitions were undertaken for the “ideal” interface and 12 for each of the socket conditions). Activation of the hand was detected using the electronic goniometer.

Full details of all these assessments are provided in Chadwell et al.^18^.

#### Electromechanical delay

A simple bench-top method was used to measure the electromechanical delay between an artificially generated activation signal applied at the electrode and initial movement of the prosthetic hand.

**Figure 4:**
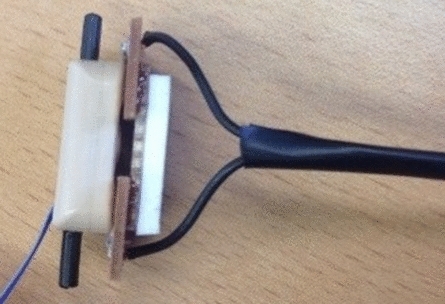
To provide good contact with the electrodes for the measurement of the electromechanical delay, the researcher held a piece of stripboard against the electrodes. A piece of foam was used provide insulation between the researcher’s fingers/thumb and the electrodes. Although this image shows how the stripboard would ideally contact the electrodes, for the assessment of electromechanical delay, the electrodes were embedded in the socket as shown in Fig. 2.

To generate the artificial activation signal, the two outer electrodes of the myoelectrode were contacted by short wires connected through a fast-acting relay. To provide good contact between the electrodes and the circuit, stripboard was used (Fig. 4). Further figures describing the circuit are provided in the supplementary documentation. When the switch was opened this generated a difference in the voltage on each wire, activating the prosthesis; whilst closed, the voltage was the same at each electrode. The switch was controlled via an Arduino which also controlled the collection of data from a goniometer placed across the proximal knuckle of the index finger. The goniometer data was sampled at 1000 Hz and filtered using a 2nd order double-pass Butterworth filter with a cut-off frequency of 20 Hz. By measuring the time between the start of goniometer recording (synchronised with the switch activating) and the onset of hand opening/closing (angle exceeded 1° above or below the mean resting value calculated from the first 80 ms) it was possible to quantify the delay.

The delay to the onset of hand opening was measured from a fully closed aperture and from a neutral aperture, and the delay to the onset of hand closing was measured from a fully open aperture and from a neutral aperture (5 times each, total = 20 measurements).

To measure the delay to open the hand, the circuit was first placed in contact with the ‘open’ electrode with the switch closed. The ‘close’ electrode was activated by touching it with a finger to set the hand in the required starting position (closed or neutral aperture). Once the hand was in the starting position, a signal was sent from the computer to an Arduino to begin the assessment, activating the hand by opening the switch, and measuring the delay between the switch opening and the onset of hand movement.

Initial pilot work suggested that the delay to movement onset when the hand begins fully closed, is longer than the delay from any other hand aperture. It is believed that the difference is due to stiction between parts designed to move relative to one another, backlash in the gears, and some give in the metal of the finger and thumb when the hand is fully closed. For more information see the supplementary material, where a short study is presented to evaluate the delay to movement onset from different starting hand apertures.

### Protocol for the assessment of user performance

#### Functionality

**Figure 5:**
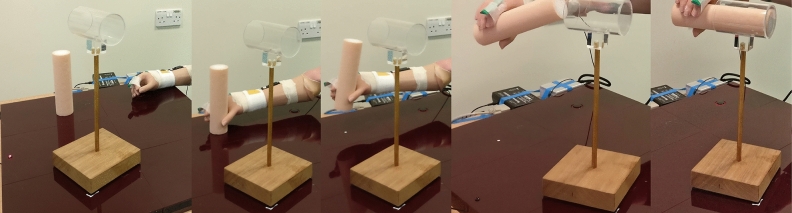
Cylinder task, participants were asked to reach to grasp a nylon cylinder with a foam outer surface (diameter 52 mm, length 200 mm, weight 350 g). Lift it and rotate it to the horizontal before placing it into a transparent tube (inner diameter 64 mm, length 100 mm).

Participants were asked to perform a multi-stage functional task where they were to reach to grasp a cylinder, lift and rotate it, and place it into a horizontal tube^18^ (Fig. 5). All participants attempted the cylinder task 10 times.

An electronic goniometer (Biometrics Ltd) was worn across the proximal joint of the index finger, an Inertial Measurement Unit (Xsens MTw) was worn just proximal to the wrist on the rear of the forearm, and a Dikablis Professional Wireless Eye Tracker system (Ergoneers) was worn to track eye movements. The goniometer and Inertial Measurement Unit were sampled at 50 Hz, the field of view camera for the eye tracker was sampled at 30 Hz, and the eye cameras were sampled at 60 Hz. The three systems were synchronised via an arcade style button and an LED (Fig. 5 shows the arcade button beneath the hand’s start position, and the LED at the top of the board—far left of 1st image). The LED also acted as a focal point for the participant at the start of each trial.

**Figure 6:**
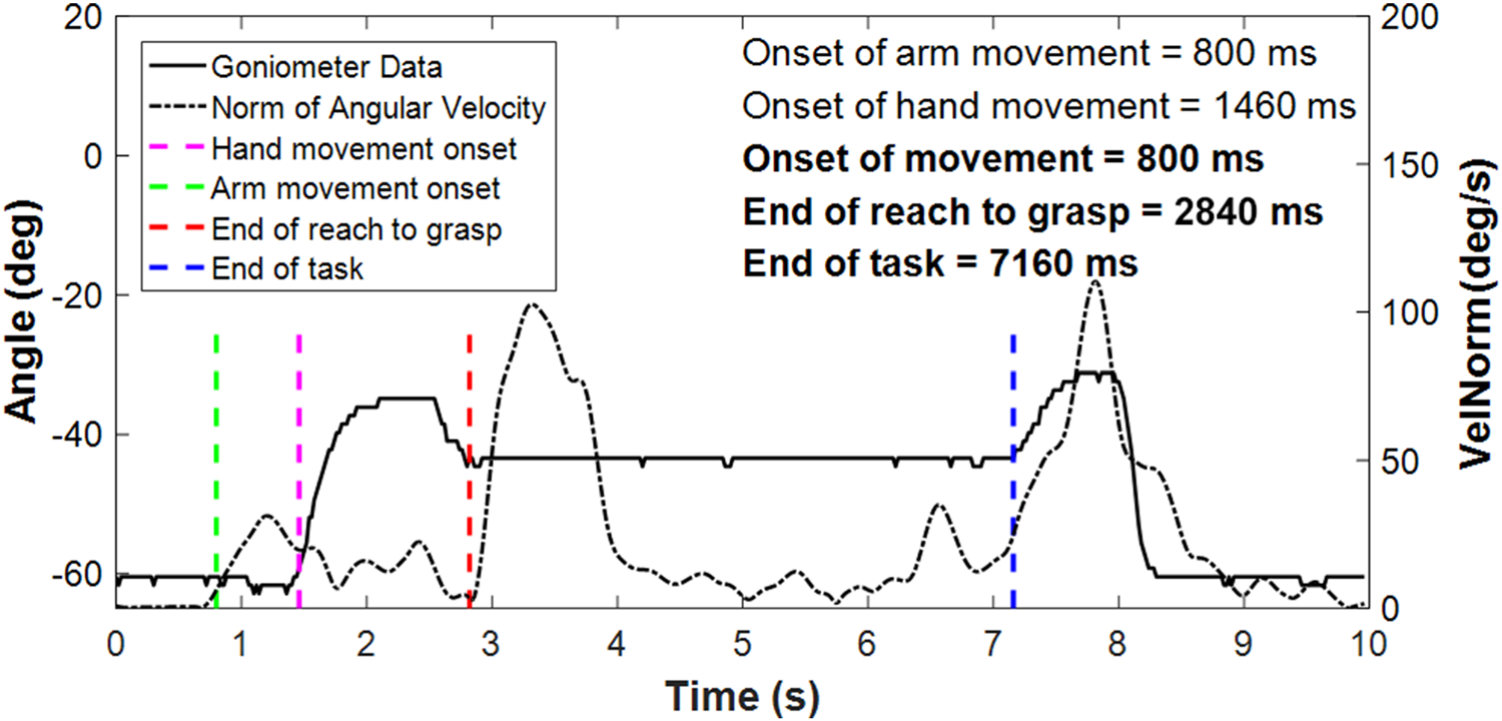
Example data and the timestamps identified by the algorithms. This example shows a smooth completion of the task. The bold solid line shows the angle data recorded by the goniometer worn across the proximal knuckle of the index finger, the dashed line shows the norm of the angular velocity recorded from the IMU worn on the wrist. The coloured vertical lines show the automatically detected segmentation points. The onset of arm movement is shown in green, the onset of hand opening is highlighted in magenta, the end of reach to grasp is shown in red, and task completion in blue.

Figure 6 shows the automatically identified segmentation points for a typical smoothly performed task. The goniometer data (solid line) shows the hand opening and closing around the object, then opening again to release the object. Participants were asked to start and end the task with the hand closed. The norm of the angular velocity (dashed line) shows an initial peak as the forearm is lifted from the tabletop, a significantly larger peak as the forearm is rotated to move the cylinder into a horizontal orientation, and a final large peak as the forearm is rotated back into a comfortable orientation after releasing the cylinder. These distinctive features would not occur if the participant were permitted to use a wrist rotator. Task onset was taken as the first point of movement, either the onset of hand opening or the first moment of forearm lift. The onset of hand opening was defined as the 1st point where the goniometer angle exceeded the Mean Resting Value from the 1st 500 ms by 1° and continued to increase by ≥ 5° over the next 200 ms. The first moment of forearm lift was defined as the 1st point at which the norm of the angular velocity of the forearm exceeded 5.73°/s. The end of reach-to-grasp was identified as the 1st point where the hand closing speed reduced to 2.5°/s. Task completion was identified as the 1st point after the “transport plateau” where the goniometer angle exceeded the Mean Resting Value during the “transport plateau” plus 1 standard deviation (the participant had begun to release the cylinder). See appendix 5 of Chadwell’s thesis^20^ for more details on the choice of thresholds.

User functionality was evaluated based on:

- Task success—The number of trials successfully completed (out of 10). Only half a point was awarded if the task was not completed in one smooth movement e.g. the hand opened and closed more than once during reach-to-grasp, or if the hand released the cylinder before it was all the way into the tube (provided the cylinder was placed far enough into the tube that it remained there after release). Goniometer data was analysed using a Matlab program; signal plots and video data from the eye tracker were also visually inspected. If any trial was deemed incomplete (e.g. if the cylinder was knocked over or dropped) and received no points, it was excluded from all further functionality measures. Higher success rates were assumed to correspond to higher functionality.
- Task duration—The mean duration across the 10 trials; it was assumed that the faster the task was completed, the higher the functionality. Many existing upper-limb outcome measures such as the Box and Blocks or the Southampton Hand Assessment Procedure assess functionality based on performance time.
- Reach aperture plateau length—Prosthesis users have previously been shown to demonstrate a plateau between the opening and closing of the hand during reach-to-grasp representing a decoupling of reach and grasp; longer plateaus have been shown to correspond to lower functionality^22^. Figure 6 shows the goniometer data (solid black line) and the plateau can be seen between the 2nd and 3rd vertical dashed lines. The algorithm to identify the plateau first finds the maximum hand aperture, and then defines a threshold value 2° below this maximum. The period during which the aperture exceeded this threshold was taken as the plateau length. The length of the plateau is expressed as a percentage of the reach-to-grasp phase (mean of 10 trials).
- Movement variability—The measures developed by Thies et al.^23^ were used to calculate the mean trial-trial, temporal variability in the patterns of acceleration measured at the wrist of the prosthesis. This involves non-linear dynamic time-warping of each pair of acceleration signals, one from each trial, to calculate the amount of warping needed to most closely align one trial onto the other. The resulting value for each pair of the successful trials, is expressed as a non-dimensional value, the warp cost^23^. Finally, the average of the warp costs for each pairing of the trials is calculated, as a measure of the trial-trial variability across the successful attempts. Higher warp cost has been shown to be associated with lower functionality^24^.
- Gaze patterns—Prosthesis users often rely on visual feedback of the hand state (open/closed)^25–28^. During multi-stage tasks, lower prosthesis user functionality has been hypothesised to be associated with more time spent looking at the hand and less time looking ahead to future components of the task^26^; however, data suggests gaze behaviour may also be influenced by other features^26,27^. Patterns of gaze recorded included the percentage of time spent looking at different areas of interest and the number of switches in gaze focus between different areas of interest—typical for monitoring the hand state as well as the progress towards the target (mean of 10 trials). During the reach-to-grasp phase the task area was grouped into 3 areas of interest: time spent looking at the hand, time spent looking at the grasp critical area (GCA) of the cylinder (the lower half), and time spent looking at the location critical area (LCA) of the cylinder (the upper half) and/or the tube. During the transport phase the task area was grouped into 2 areas of interest: time spent looking at the hand and/or the GCA of the cylinder, and time spent looking at the location critical area (LCA) of the cylinder and/or the tube. Further guidelines on the definition of these areas of interest and the results of an inter-rater reliability study to assess the coding guidelines are provided as supplementary material.

#### Everyday prosthesis usage

To evaluate actual usage of the prosthesis outside of the clinic, participants wore an Actigraph activity monitoring sensor (tri-axial accelerometer) from the GT3X range, on each wrist over the period of 7 days. Using proprietary Actilife software, the raw acceleration was filtered, grouped into epochs, and converted into activity counts^29^. In this study we were focussed on the resultant vector magnitude of the activity counts across all three axes for each 60 s epoch. Using these vector magnitude values, the percentage reliance on the anatomically intact arm was calculated for each epoch of activity data using the following equation:$\mathrm{\%}{Reliance}_{Anatomical}= \left({VM}_{Anatomical}/\left({VM}_{Anatomical}+{VM}_{Prosthesis}\right)\right)\times 100$. The median percentage reliance across all 7 days was reported. Prosthesis wear time was calculated using a non-wear algorithm published in Chadwell et al.^30^ The time spent using each of the arms unilaterally and the ratio of unilateral activity between the two arms (Anatomical:Prosthesis) are also reported. In our previous study^17^ we found no relationship between our measures of functionality and everyday prosthesis usage, but it is reasonable to assume that more daily wear and increased symmetry of upper-limb activity may be correlated with higher levels of EMG skill, lower unpredictability, and shorter electromechanical delays.

### Statistical analysis

The correlation analysis presented in this article uses Kendall’s tau-b with 2-tailed testing. Kendall’s tau-b was chosen as it lends itself to small sample sizes and data with tied ranks^31^. It is worth noting that this analysis is exploratory, and by undertaking several correlation tests, the multiple comparisons problem means that there is an increased likelihood of Type 1 error. This means that when many statistical tests are performed, some are expected to give significant results by chance. This analysis aims to highlight potential relationships to guide future more targeted studies. To enable readers to further explore these relationships, for correlations τ_b_ > 0.3 or τ_b_ < − 0.3 (regardless of p-value), we have provided scatter plots as supplementary material.

## Results

To allow for later comparisons, first the prosthesis users’ performance will be described. This includes both their functionality and their everyday prosthesis usage. Their ability to control the prosthesis will then be described and the correlations between these control factors and user performance will be presented.

### Prosthesis user performance

A wide range of scores were recorded for all performance measures between the participants.

#### Functionality

##### Task success rate and duration

Nine participants had a success rate of 10/10, a further nine participants had a success rate of 7.5–9.5. Only two participants showed significant difficulty in completing the task (success rate ≤ 3.5); both struggled with unexpected hand opening while attempting to rotate the grasped cylinder to the horizontal prior to placing it into the tube, and one never managed to get the cylinder all the way into the tube before the hand unexpectedly released it. The grand mean task duration for the successful trials was 5.87 s (min = 2.7 s, max = 9.3 s, SD = 1.98 s).

##### Patterns in hand aperture

Six participants demonstrated some difficulty either opening the hand or closing it around the cylinder during 1–4 of their trials. The grand mean length of the reach plateau (between the opening and closing of the hand) for the successful trials was 29% of the reach-to-grasp phase (min = 13%, max = 43%, SD = 9%).

##### Movement variability

The warp cost for the successful trials ranged from 7.59 to 55.69 (median = 18.90, Q1 = 13.84, Q3 = 27.31).

##### Gaze patterns

**Figure 7:**
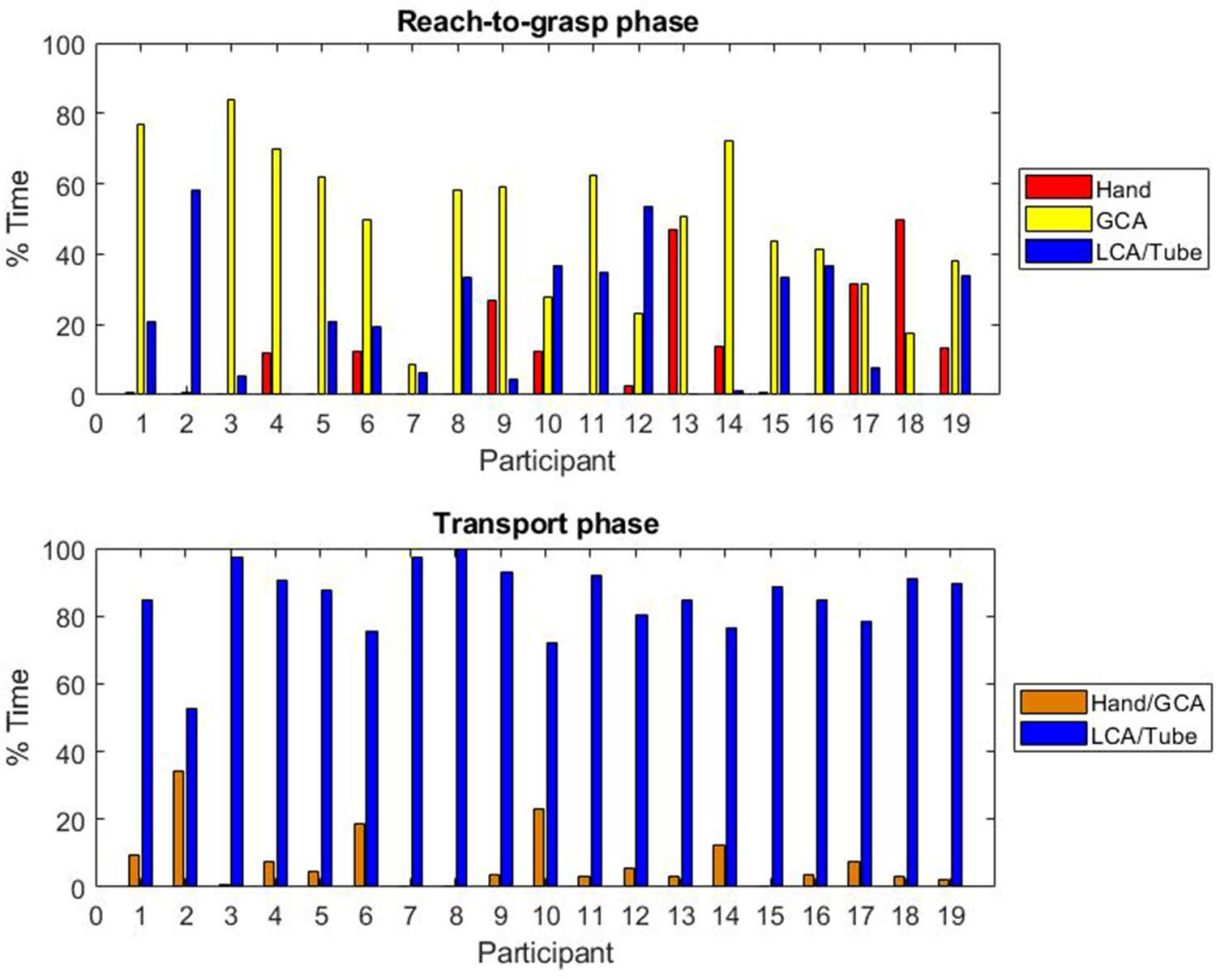
Mean time spent looking at each Area of Interest as a percentage of the reach-to-grasp and transport phases. Missing data and time spent looking at parts of the task other than the hand, cylinder and tube are not displayed. Gaze data was only recorded for 19 participants. GCA = Grasp critical area of cylinder, LCA = Location critical area of cylinder.

Gaze data was only available for 19 participants due to poor calibration for one participant. Larger between subject differences were noted for the reach-to-grasp phase than the transport phase (Fig. 7 shows the mean time spent looking at each area of interest).

During reach-to-grasp, only five participants did not look at the hand at all; the maximum time spent looking at the hand was 50% of the reach-to-grasp phase (median = 3%, Q1 = 0%, Q3 = 14%). All participants spent some time looking at the cylinder and/or tube whilst performing reach-to-grasp; the time spent looking at the grasp critical area (GCA) ranged from 1 to 84% of the reach-to-grasp phase (median = 50%, Q1 = 28%, Q3 = 62%), and the time spent looking ahead to the location critical area (LCA) and/or tube ranged from 0 to 58% of the reach-to-grasp phase (median = 21%, Q1 = 4%, Q3 = 35%).

During the transport phase, only two of the participants did not look at the hand at all (only one of these two participants did not look at the hand at all throughout both parts of the task). All participants spent more time looking at the LCA and/or tube, than at the hand and/or GCA. The time spent looking at the hand and/or GCA ranged from 0 to 34% of the transport phase (median = 4%, Q1 = 2%, Q3 = 10%), whilst the time spent looking at the LCA and/or tube ranged from 52 to 100% of the transport phase (median = 88%, Q1 = 78%, Q3 = 92%).

The mean number of gaze switches for each participant during the reach-to-grasp and transport phases respectively ranged from 2 to 21 (median = 5, Q1 = 3, Q3 = 8) and from 0 to 12 (median = 4, Q1 = 3, Q3 = 6). However, the large majority of these involved a transition to look at something other than the hand, GCA, LCA, or tube (reach-to-grasp phase: median = 71%, IQR = 32%, transport phase: median = 79%, IQR = 33%). During reach-to-grasp six participants performed a mean of 1 or more gaze switches between the hand and the GCA (max = 11), and four other participants performed a mean of 1 or more switches between the GCA and the LCA and/or tube (max = 3). All participants performed fewer than 1 switch (mean) between the hand and the LCA and/or tube. During the transport phase seven participants performed a mean of 1 or more switches between the hand/GCA and the LCA/tube (max = 2).

#### Everyday prosthesis usage

Prosthesis wear time over the 7 days ranged from 3–107 h (median = 46 h, Q1 = 25 h, Q3 = 89 h). During the periods the prosthesis was worn the median percentage reliance on the anatomical arm ranged from 67–87% (median = 80%, Q1 = 74%, Q3 = 85%). The ratio between the time spent using the anatomical arm unilaterally (min = 23 min, max = 732 min, median = 189 min) and the time spent using the prosthetic arm unilaterally (min = 0 min, max = 82 min, median = 15 min) ranged from 4.3:1 to 73:1 (median = 11.5:1). Two participants did not use the prosthesis unilaterally at all throughout the recording period resulting in infinite unilateral activity ratios.

### Control factors and their relationships with user performance

#### EMG skill

##### Intuition task

The group median SRT was 308 ms for opening the hand and 312 ms for closing the hand. The group median CRT was slightly higher at 385 ms for opening the hand, and 381 ms for closing the hand.

For three of the participants the decision time (CRT-SRT) to open the hand was negative (meaning the mean SRT was longer than the mean CRT), and for one of these three participants the decision time to close the hand was also negative suggesting there was a learning effect. The median decision time to open the hand across participants was 58 ms, whilst the median decision time to close the hand was 62 ms. We hypothesised that shorter decision times would be correlated with higher levels of prosthesis user performance. Both the decision time to open the hand (τ_b_ = 0.357, p = 0.033, n = 19) and the decision time to close the hand (τ_b_ = 0.333, p = 0.046, n = 19) were correlated with the time spent looking at the location critical area and/or the tube during reach. People who took longer deciding which muscle to activate spent a larger percentage of the reach phase looking ahead to the location critical area of the cylinder or at the tube. This was contrary to our hypothesis. The scatter plots of these relationships are provided in the supplementary material (Fig. 27). No other significant correlations with τ_b_ < − 0.3 or τ_b_ > 0.3 were found.

##### Static tracking task

**Figure 8:**
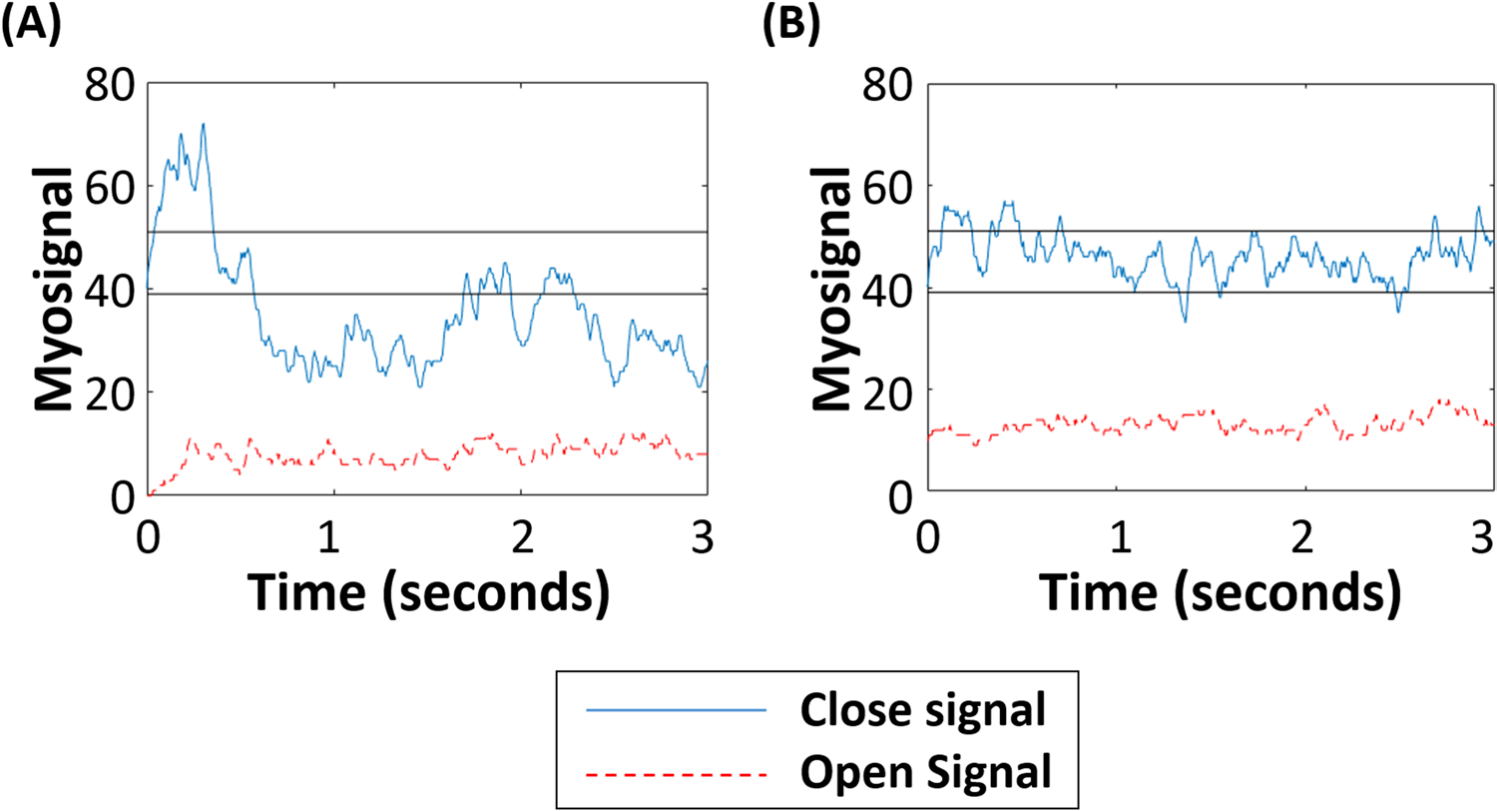
Static Tracking Task closing trial example for the (**A**) worst (22%) and (**B**) best (85%) performers. Participants were instructed to keep the blue signal (controlled using the muscle for hand closing) inside the boundary lines, whilst keeping the other muscle (red dotted) relaxed. The trial lasted 3 s.

The results showed clear differences between participants. For the muscle used to open the hand (wrist flexor), the median score was 54% of the signal kept within the boundaries (min 23%, max 74%, IQR 22%), and for the muscle used to close the hand (wrist extensor), the median score was 59% (min 22%, max 85%, IQR 25%). Figure 8 shows an example of (A) the minimum (22%) and (B) the maximum (85%) wrist flexor (hand closing) attempts. We hypothesised that participants who were better able to control the contraction level of their muscles would demonstrate higher functionality and everyday prosthesis usage, however, there were no significant correlations between the performance on the static tracking task and any of the measures of performance.

##### Dynamic tracking task

For the first part of the task, controlling one car using the wrist extensor (open) signal the median pass rate was 80% (IQR = 13%), and for the wrist flexor (close) signal the median pass rate was 76% (IQR = 13%). For the second part of the task, controlling two cars simultaneously (one with each muscle), on average the pass rates were lower. For the wrist extensor (open) signal the median pass rate was 66% (IQR = 31%), and for the wrist flexor (close) signal the median pass rate was 59% (IQR = 40%). It is worth noting that some participants struggled to complete the two-car task; two self-reported struggling to visually track both cars on the screen at once, whilst three others were unable to activate one of their muscles without also contracting the antagonistic muscle. The other 15 participants performed better when only given one car to control than when attempting to control both cars simultaneously.

With respect to correlations between the dynamic tracking task and the measures of user performance, we hypothesised that participants with better control over the amplitude of their muscle signals would demonstrate higher levels of functionality and everyday prosthesis usage. We found participants who performed better on the single car task using the muscle signal for hand opening took longer to perform the functional task (τ_b_ = 0.332, p = 0.044, n = 20). This was contrary to our hypothesis. The scatter plots of these relationships are provided in the supplementary material (Fig. 27).

Participants who performed better on the two car task using the muscle signal for hand closing demonstrated more symmetrical everyday arm use with reduced reliance on their anatomically intact side (τ_b_ = − 0.339, p = 0.019, n = 20).

No other significant correlations with τ_b_ < − 0.3 or τ_b_ > 0.3 were found.

#### Unpredictability

##### Desired activation

For twelve of the participants, the difference in the spread of reaction times when closing the hand was positive (max difference = 158 ms); only eight participants showed a positive difference in spread for the open movement (max difference = 138 ms). For 14/20 participants the difference in spread was negative for one or both of opening/closing.

The difference in the spread of reaction times to open the hand was correlated with the length of the reach plateau (constituting a separation of reach and grasp). A larger spread corresponded to a shorter reach plateau (τ_b_ = − 0.368, p = 0.023, n = 20). No significant correlation was found for closing the hand.

For some trials, the prosthesis did not respond at all, or responded incorrectly. Across the twenty participants, 800 reaction trials were undertaken using the “ideal” interface (769 successful), and a further 800 trials were undertaken using one or other of the two socket conditions (400 without additional load and 400 with additional 500 g load) (693 successful). A note was kept if the participant believed that they were attempting to make the correct response.

One participant struggled to operate the hand using the “ideal” interface; from discussions with the participant it is possible that the lack of a prosthetic socket was causing them confusion as to how to operate the hand (‘overthinking’), and they therefore regularly responded too slowly or with the incorrect muscle activation. For the other 19 participants, everyone completed over 38/40 responses correctly for the “ideal” interface condition. Eight participants successfully completed 39 or 40 responses (out of 40) for both conditions (“ideal” and socket), and the other eleven participants completed a lower number of trials successfully during the socket conditions than with the “ideal” interface (min = 2 fewer successful trials, Q1 = 2, median = 7, Q3 = 10, max = 17). In the socket conditions some participants were unable to operate the hand at all with their arm in the + /- 45° positions, this is reflected in a low number of successful responses e.g. one participant completed none of the 10 closing trials with the arm held at a − 45° angle as they were unable to close the hand in that position without pressing the socket (and electrodes) against the arm with their anatomical hand.

We hypothesised that participants whose hand responded correctly to their muscle signals most of the time would demonstrate higher functionality and everyday prosthesis usage. Using the “ideal” interface we found no significant correlations with τb < − 0.3 or τb > 0.3. When wearing their own prosthetic socket, we found that participants who responded correctly to fewer open trials demonstrated higher kinematic variability (τ_b_ = − 0.487, p = 0.005, n = 20) and spent a higher percentage of the reach-to-grasp phase looking ahead to the location critical area and/or the tube (τ_b_ = − 0.431, p = 0.015, n = 19).

##### Undesired activation

Participants undertook several trials where they moved the arm between 45° above and below the horizontal without attempting to activate the hand (or to resist undesired activations). Using the “ideal” interface to perform the task, across all 20 participants, eleven undesired activations of the hand occurred (out of 480 transitions), eight of these were undesired hand closing, and seven were recorded from only two of the participants. For the socket conditions, 56 undesired activations occurred (out of 480 transitions); 29 of these were undesired opening of the hand, the remaining 27 were undesired closing. Sixteen of the participants experienced at least 1 undesired activation of the hand across all conditions (min = 0, Q1 = 1, median = 2.5, Q3 = 5, max = 16).

This factor showed the strongest relationship with the measures of functionality (see Table 1). Higher numbers of undesired activations of the hand were significantly correlated with a lower success rate, longer task duration, higher temporal kinematic variability, more time spent looking at the hand, less time spent looking at the grasp critical area during reach-to-grasp, and more gaze switches. It is worth noting that in the “ideal” skin–electrode interface condition many participants experienced no undesired activations of their hand which is reflected in the scatter plots (see supplementary material).

**Table 1:** Kendall’s tau-b correlation matrix (2-tailed), comparing the total number of undesired hand activations to the measures of functionality on the cylinder task.

		Success Rate	Duration	Reach plateau length	Kinematic variability	Gaze Time Hand (Reach)	Gaze Time GCA (Reach)	Gaze Time LCA/Tube (Reach)	Gaze Time Hand/GCA (Transport)	Gaze Time LCA/Tube (Transport)	Total number of gaze switches
“Ideal” skin–electrode interface	No. undesired open	τ = − .067 p = .741 n = 20	τ = .036 p = .850 n = 20	τ = .370 p = .051 n = 20	τ = .131 p = .489 n = 20	τ = − .093 p = .640 n = 19	τ = − .375 p = .055 n = 19	τ = .427* p = .029 n = 19	τ = .272 p = .164 n = 19	τ = − .323 p = .098 n = 19	τ = .168 p = .389 n = 19
	No. undesired close	τ = − .064 p = .750 n = 20	τ = .237 p = .209 n = 20	τ = − .155 p = .411 n = 20	τ = .286 p = .130 n = 20	τ = .110 p = .578 n = 19	τ = − .249 p = .199 n = 19	τ = .231 p = .233 n = 19	τ = .160 p = .408 n = 19	τ = − .160 p = .409 n = 19	τ = .356 p = .066 n = 19
	Total no. undesired activations	τ = − .169 p = .394 n = 20	τ = .293 p = .114 n = 20	τ = − .038 p = .839 n = 20	τ = .369* p = .047 n = 20	τ = .059 p = .761 n = 19	τ = − .385* p = .043 n = 19	τ = .369 p = .053 n = 19	τ = .271 p = .155 n = 19	τ = − .287 p = .132 n = 19	τ = .385* p = .043 n = 19
Own socket (incl. trials with + 500 g load)	No. undesired open	τ = − .521** p = .006 n = 20	τ = .589** p = .001 n = 20	τ = − .019 p = .915 n = 20	τ = .463** p = .009 n = 20	τ = .357 p = .054 n = 19	τ = − .249 p = .170 n = 19	τ = .083 p = .647 n = 19	τ = .056 p = .760 n = 19	τ = − .305 p = .093 n = 19	τ = .415* p = .022 n = 19
	No. undesired close	τ = − .220 p = .250 n = 20	τ = .316 p = .078 n = 20	τ = .038 p = .832 n = 20	τ = .404* p = .024 n = 20	τ = .051 p = .786 n = 19	τ = − .221 p = .233 n = 19	τ = .050 p = .787 n = 19	τ = .086 p = .644 n = 19	τ = .007 p = .969 n = 19	τ = .121 p = .513 n = 19
	Total no. undesired activations	τ = − .537** p = .003 n = 20	τ = .612** p = < .001 n = 20	τ = .095 p = .574 n = 20	τ = .522** p = .002 n = 20	τ = .322 p = .071 n = 19	τ = − .250 p = .153 n = 19	τ = .000 p = 1.000 n = 19	τ = .132 p = .452 n = 19	τ = − .187 p = .283 n = 19	τ = .387* p = .027 n = 19
Across all conditions	Total no. undesired activations	τ = − .504** p = .005 n = 20	τ = .631** p = < .001 n = 20	τ = .155 p = .358 n = 20	τ = .553** p = .001 n = 20	τ = .347* p = .049 n = 19	τ = − .343* p = .047 n = 19	τ = .147 p = .396 n = 19	τ = .252 p = .146 n = 19	τ = − .318 p = .066 n = 19	τ = .465** p = .007 n = 19

** Correlation is significant at the .01 level. *Correlation is significant at the .05 level.

Fewer relevant significant correlations were seen with the everyday usage measures. Participants with more undesired closing of the prosthesis using the “ideal” electrode–skin interface showed less symmetrical upper-limb activity (τ_b_ = 0.318, p = 0.046, n = 20). Further, participants whose own socket led to large numbers of undesired closing of the prosthesis tended to wear the prosthesis more (τ_b_ = 0.404, p = 0.024, n = 20), which was contrary to our hypothesis. This may suggest that other factors such as cosmesis may affect the real-world use of the prosthesis.

#### Delays

The electromechanical delay in the prosthesis between electrode activation and the onset of hand movement was recorded for 14 of the prostheses. We were unable to record any of the measures of delay for the remaining six prostheses; reasons for missing data included an older style of electrodes which were a different shape and hence not compatible with the setup, a wrist rotator which the participant was unable to turn off, and material on the inside of the socket which prevented good contact with the electrodes. For Prosthesis 1, we were unable to set the hand in a neutral position whilst the equipment was set up to activate the open electrode; the delay to open from neutral was therefore not taken for this prosthesis.

**Figure 9:**
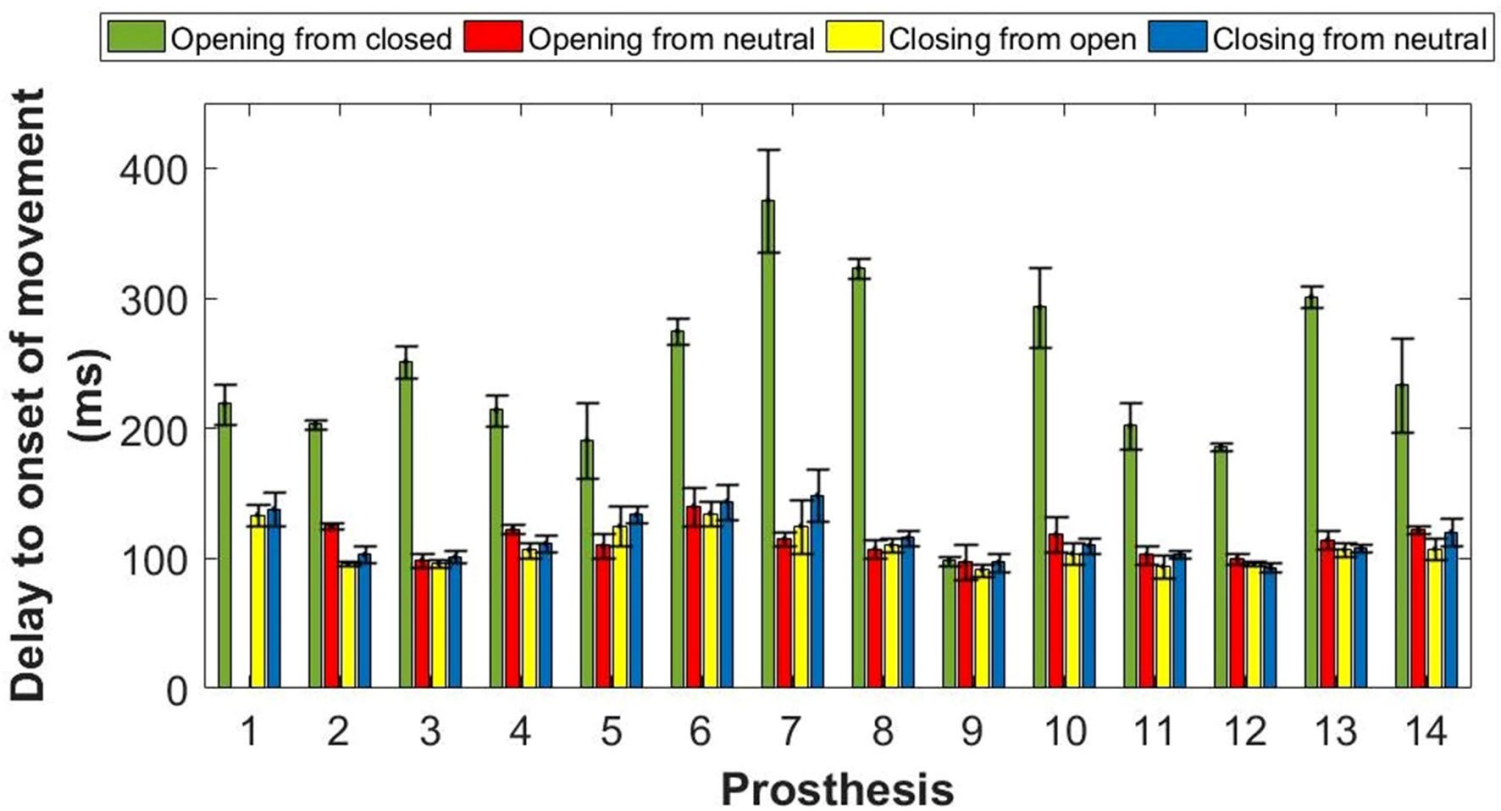
Mean (± SD) delay (out of 5 trials) to the onset of hand movement for each of the 14 user owned prostheses. For all hands except hand 9, the delay to the onset of hand opening from a fully closed position was significantly longer (t-test: p ≤ 0.01) than all other measures of delay. For hand 1, it was not possible to record the delay to the onset of hand opening from a neutral aperture.

Across all of the prostheses, the grand mean delays and standard deviations were 240 ± 69 ms to the onset of opening from a closed position, 116 ± 17 ms to the onset of opening from a neutral position, 109 ± 15 ms to the onset of closing from an open position, and 116 ± 18 ms to the onset of closing from a neutral position. For 13 of the 14 assessed prostheses the delay to the onset of opening from a closed position was significantly longer (t-test p ≤ 0.01) than the delay from a neutral position, or the delays to the onset of hand closing (Fig. 9).

We predicted that a shorter electromechanical delay would correspond with higher functionality and everyday prosthesis usage. We found significant correlations with task duration, the length of the aperture plateau, the gaze measures during the transport phase, and prosthesis wear time (Table 2).

**Table 2:** Kendall’s tau-b correlation matrix (2-tailed), comparing the electromechanical delay in the prosthesis to a sub-selection of the outcome measures.

	Duration	Reach plateau length	Gaze Time Hand/GCA (Transport)	Gaze Time LCA/Tube (Transport)	Total number of switches (Transport)	Prosthesis wear time
Delay to open from closed		τ = − .495* p = .014 n = 14		τ = .436* p = .038 n = 13		
Delay to open from neutral				τ = .455* p = .040 n = 12	τ = − .515* p = .020 n = 12	τ = .436* p = .038 n = 13
Delay to close from open	τ = − .411* p = .042 n = 14		τ = − .562** p = .008 n = 13	τ = .805** p = < .001 n = 13	τ = − .597** p = .005 n = 13	
Delay to close from neutral		τ = − .451* p = .025 n = 14	τ = − .555** p = .009 n = 13	τ = .641** p = .002 n = 13	τ = − .615** p = .003 n = 13	

Only significant correlations with τ values > 0.3 or < -0.3 are shown. ** Correlation is significant at the .01 level. *Correlation is significant at the .05 level.

## Discussion

In this study each control factor was characterised using multiple variables. Performance using the ‘open muscle’ often differed from performance using the ‘close muscle’, therefore the two measures were reported separately.

We found no significant correlations between the majority of the measures reflective of EMG skill (choosing the correct EMG signal to generate or holding the EMG signal at a prescribed level) and user performance. There were significant correlations between the time taken to decide which muscle to activate to operate the hand and a measure of gaze behaviour, between one measure of tracking an EMG signal and the time taken to complete the cylinder task, and between another EMG tracking measure and one of the measures relating to symmetry of real-world arm use, however, these correlations were fairly weak (τ_b_ = 0.332 to 0.357). Researchers have shown that performance on a given myoelectric task does not correlate with performance on a different myoelectric task, suggesting that a general myocontrol skill does not exist^13^. Another study from the same group found no relationship between skill in controlling EMG and performance on a functional task^10^. Our findings tend to support this finding that the degree of skill in controlling EMG may not be a key factor in determining performance on functional tasks. Further, our study found little substantial evidence of a relationship between skill in controlling EMG and patterns of real-world prosthesis use.

The factors most correlated with user functionality were those related to the unpredictability of the hand response. These included both the number of undesired activations when the user did not actively input a control signal, and the times when the hand either did not respond or responded incorrectly to a control signal. Interestingly however, the predictability of the prosthesis response had little correlation with how much the participant chose to wear and or use the prosthesis on a day-to-day basis. This is an important finding, as it suggests there are many context-specific decisions affecting a person’s decision to use their prosthesis beyond their ability to control the device.

Participants with more undesired responses of the prosthesis were less successful at completing the cylinder task. This was generally reflected in difficulty smoothly performing a reach-to-grasp, or by the hand releasing the cylinder prematurely as it was rotated to a horizontal position (Note that this motion of the forearm results in the electrode positions moving to be above/below the arm increasing the risk of electrode lift-off). These issues performing the task may explain the correlation with task duration, movement variability, and fewer look-ahead gaze fixations/increased reliance on looking at the hand during reach-to-grasp or flicking focus between different areas of the task.

When transitioning the arm between a position 45° above the horizontal to a position 45° below the horizontal, 80% of participants experienced at least one undesired activation of the prosthetic hand (out of 48 transitions), and during the trials where the participant’s own prosthesis was worn on average 11.7% of transitions resulted in undesired activations of the hand (compared to 2.3% using an “ideal” electrode interface). This supports the findings of Head^14^ who presented example EMG plots for 5 participants undertaking 3 arm movements. When a load was added to the prosthesis all participants in Head’s study demonstrated EMG signals above the threshold to activate the hand and only one participant was able to perform a reach movement with their prosthesis without an undesired signal being generated. Similarly, in Monk’s thesis^32^ where 6 participants undertook 3 similar arm movements using three different socket designs, participants experienced more undesired hand activations when wearing a prosthetic socket (65–75% of transitions) than when using an “ideal” skin–electrode interface (30% of transitions). The data presented in our study, collected using participants’ own prostheses, illustrates well how far current myoelectric prostheses are from offering the almost perfect predictability of hand response anatomically intact people take for granted.

Furthermore, during the reaction time task designed to assess the desired activation of the prosthesis, only six participants managed to complete all 40 trials successfully when wearing their own prosthesis, with eight participants failing to complete more than a quarter of the trials. Where failed trials involved an incorrect response, it was not possible to differentiate between an incorrect response by the user and an incorrect response of the hand. However, interestingly two participants reported that when concentrating on the reaction time LED the hand would respond in a manner contrary to their intention. For example, if they intended to open their hand it would close; surprisingly when these participants looked at the hand it would respond how they desired. There is no clear explanation as to why this occurred. For one participant this phenomena occurred when using the “ideal” electrode interface and it is therefore possible that for some users, the feeling of the socket against the skin may impact their ability to generate the correct muscle contractions for prosthesis operation in the absence of visual feedback.

Another important finding from this data was that for 13/14 prostheses assessed the electromechanical delay in the prosthesis was twice as long from a fully closed position than when measured from any other hand aperture. It is therefore important to note that electromechanical delay should not be represented by a single number. The only prosthesis not to demonstrate this longer delay from a closed position was number 9 (see Fig. 9). Most participants were unaware of the exact model of hand and its setup, however prosthesis 9 behaved similarly to a threshold-controlled Steeper Select hand tested during piloting (see supplementary material). The participant was unable to inform as to whether the hand employed threshold or proportional control, however, the hand was manufactured by Steeper, and therefore it is possible that it may have been the same hand. On average, aside from the delay to open from a closed position, measured electromechanical delays were within the optimal controller delay range proposed by Farrell^16^ of 100–125 ms and varied between participants’ own devices to a relatively small (but consistent) degree. It is worth highlighting that the length of the electromechanical delay did not correlate with the participants’ functionality in the hypothesised manner. Indeed, strong correlations with some functionality measures were observed in the opposite to the expected direction, with shorter delays correlating with lower levels of functionality. It is worth noting that the impact of delays on prosthesis user performance is an underexplored area. It would be interesting to explore in future, whether the length of the delay interacts with the unpredictability measures, contributing to the relationship between unpredictability and functionality.

Previous research^25,26^ has demonstrated the disruption seen in visuomotor performance when using a prosthesis when compared to an anatomical hand. However, the level of disruption has not yet been reliably correlated to measures of skill. Our results may provide a preliminary indication that with currently prescribed prostheses and sockets, gaze behaviour is severely disrupted by mechanical issues such as delay and unpredictability, and these effects may override any relationship between gaze behaviour and skill level. The effects of mechanical issues appear to be complex. Undesired activations appear to pull gaze reactively toward the hand in most of our analyses, contrasting with characteristic predictive gaze behaviour observed during anatomical object manipulation. In contrast, increased electromechanical delay seems to lead to more predictive gaze behaviour characteristic of skilled movement. Why this should occur will require further research, but we may speculate that electromechanical delays and undesired activations may actually work in opposition to each other (increased delays actually reduce undesired activations). Our results nevertheless provide the first indication of the importance of mechanical issues beyond the user's control in determining their visuomotor efficiency.

## Limitations and future work

Although this study is one of the largest experimental studies of myoelectric prosthesis users, it is still underpowered. In order to explore the relative impact of each control factor on user performance (for example using multiple regression techniques), a much larger sample size would be required. The analysis undertaken in this manuscript is exploratory and should only be used as a guide. We have not formally adjusted for the multiple comparison problem and recommend that future researchers undertake more detailed studies focussing on particular areas of interest, or a significantly larger study. Large scale recruitment of prosthesis users is not a trivial task, we would therefore recommend that efforts are concentrated on establishing in more detail the reasons for unpredictable prosthesis responses. This may include movement when the user does not desire it, incorrect responses (opening rather than closing), or no response.

To guide future researchers undertaking similar studies we have produced a sample size calculation for Kendall’s tau-b (Table 3). We would recommend that similar studies should aim to recruit 40 + participants. The sample sizes presented in Table 3 were produced by simulating data from a bivariate normal distribution with a given correlation, computing Kendall’s tau-b, and doing a hypothesis test. The power was computed as the percentage of times *H*_*0*_ was rejected.

**Table 3:** Sample sizes needed for 80% power in testing Kendall’s τ_b_ correlation coefficient at the 5%, 1%, and 0.1% levels (2-sided tests).

τ_b_	N (5%)	N (1%)	N (0.1%)
0.3	40	58	69
0.4	23	33	50
0.5	15	23	30
0.6	11	15	21
0.7	8	11	15

The approach taken to the analysis in this manuscript was conservative due to the exploratory nature of the study. In future, researchers may wish to undertake more focussed analysis with directional hypotheses assessed using 1-tailed testing ^33^.

## Summary

This study set out aiming to establish whether EMG skill, unpredictability introduced at the skin–electrode interface, or the electromechanical delay in the prosthesis had the greatest impact on user functionality and everyday prosthesis usage. The small sample size meant that this study was unable to answer this question using multiple regression techniques; nevertheless, some control factors did show stronger relationships with performance than others (such as the relationship between the number of undesired responses of the hand, and the time taken to perform the functional task). The findings suggest that future efforts should be concentrated on better understanding why the prosthesis responds unpredictably, and how the electrode interface could be improved to reduce the number of undesired activations of the hand.

We also noted several significant correlations between the electromechanical delay in the prosthesis and the measures of user performance; however, these were all in a direction contrary to our hypothesis with longer delays correlating with higher levels of performance. This relationship therefore warrants further investigation. We also propose that future research explores the potential interactions between delays, unpredictability, and performance. Additionally, we have shown that for most prosthesis users, the delay to open the hand from a fully closed position is significantly longer than the delay from any other starting aperture. Further work would be recommended to establish the impact this has on user performance.

## Supplementary Information

Supplementary Information 1.

Supplementary Information 2.

Supplementary Information 3.

Supplementary Information 4.
