# Supplementary material for: Addressing unpredictability may be the key to improving performance with current clinically prescribed myoelectric prostheses
Source: Sci Rep. 2021 Feb 8;11:3300. doi: 10.1038/s41598-021-82764-6 (PMC7870859; doi:10.1038/s41598-021-82764-6)
Supplement: Supplementary file 2 — Supplementary Information 2. [file 41598_2021_82764_MOESM2_ESM.docx]

**Supplementary material to: Addressing unpredictability may be the key to improving performance with current clinically prescribed myoelectric prostheses**

**Development of the protocol for the measurement of delays**

**Authors:** Chadwell A., Kenney L., Thies S., Head J., Galpin A., Baker R.

## Background

In this study we defined the delay in the response of the myoelectric prosthesis to be the time difference between stimulus presentation and the onset of hand movement. Stimulus presentation is the first moment a differential voltage is provided to the electrodes. The onset of movement is defined as the point where the goniometer placed across the index finger has moved by 1 degree.

In order to measure the delay in the response, an experimental setup was required in which the electrodes could be artificially stimulated, and the time taken for the hand to move in response measured.

**Figure 10** shows a block diagram of the experimental setup. When a signal is received from the laptop, “*Arduino 1*” controls the task, opening the relay switch to stimulate the electrodes, and initiating data recording from the goniometer.


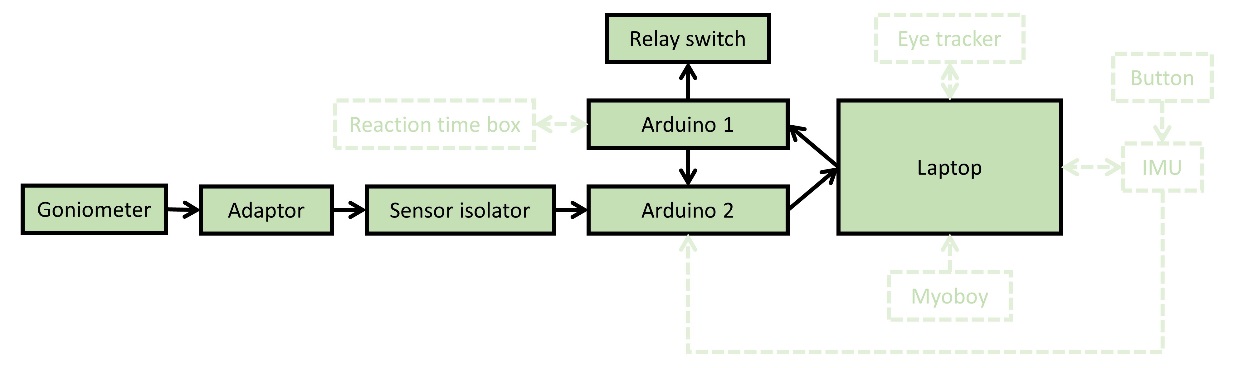


**Figure 10.** Block diagram representing the setup for the measurement of the electromechanical delay in the prosthesis response to a stimulus. Equipment from the overall experimental setup which is not included in the measurement of delays has been faded out with dotted lines. The arrows show the direction of data transfer between the hardware.

## Aims

The aims of the work were:

1. To develop an experimental setup which allowed the electrodes to be artificially stimulated.
2. To ensure the stimulation of the electrodes was synchronised with the recording of the data from the goniometer.
3. To assess whether the gain setting of the electrode impacted on the recorded delay.

And finally, pilot work suggested that the delay recorded from a closed position for one participant was longer than the reaction time for that participant, therefore the final aim of this work was:

1. To establish whether the starting hand aperture impacted on delay.

## Artificial stimulation of the electrodes

In order to activate the myoelectrode, a voltage difference between the two outer electrodes is required. Early pilot work demonstrated that it was possible to achieve this voltage difference by touching an ungrounded wire to one of the electrodes. This wire acts like an aerial with a voltage induced by electromagnetic fields in its surroundings.

In this section a simple circuit is proposed where the two outer electrodes are connected via a switch. Whilst the switch is closed the voltage across the two electrodes is the same. When the switch is open, there is a voltage difference and the electrode is activated.

### Experimental setup

#### The circuit design

The experimental setup for the artificial stimulation of the electrodes is shown in **Figure 11**. To ensure good contact with the electrodes, two flat conductive plates (stripboard) are used. These are connected to the normally closed poles of a relay switch using 10cm lengths of copper wire. A piece of insulating foam is placed over the stripboard to allow the plates to be held against the electrodes during the test without the conductivity of the finger affecting the voltage induced in the plates.


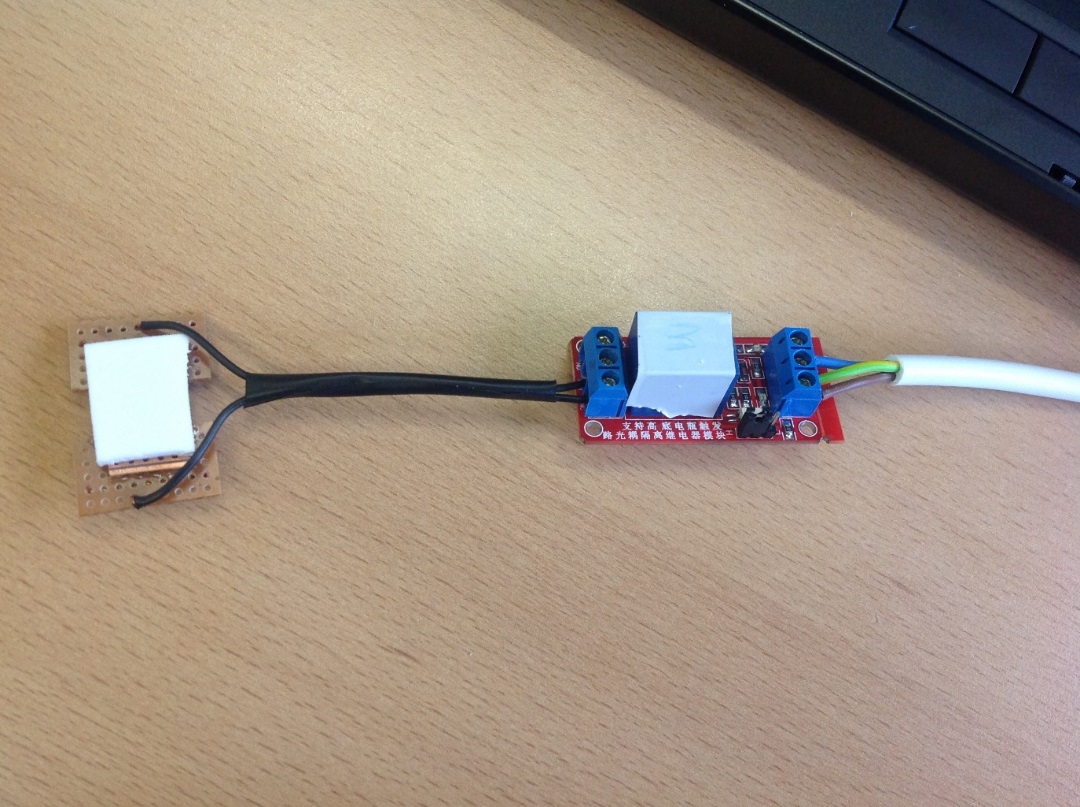

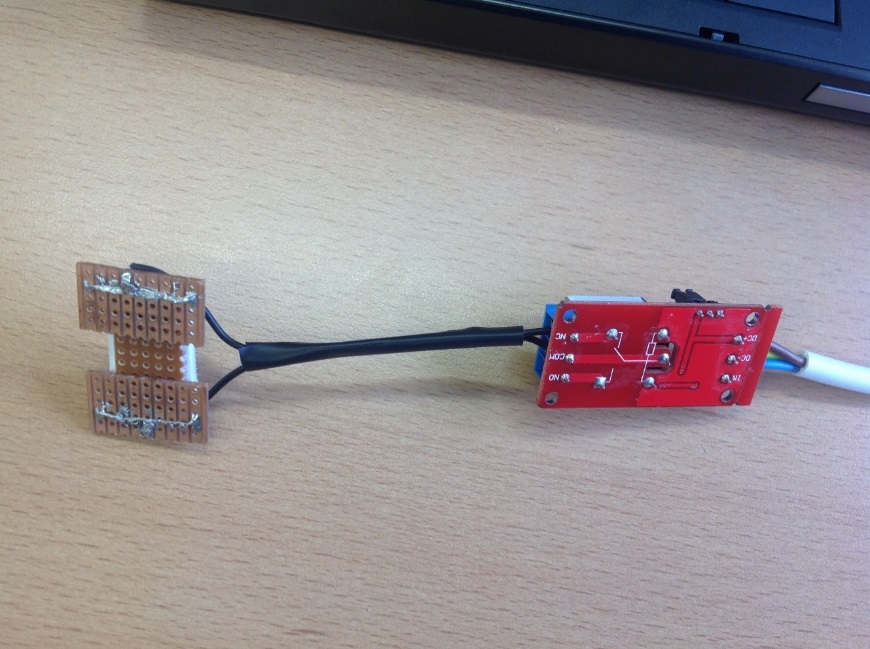

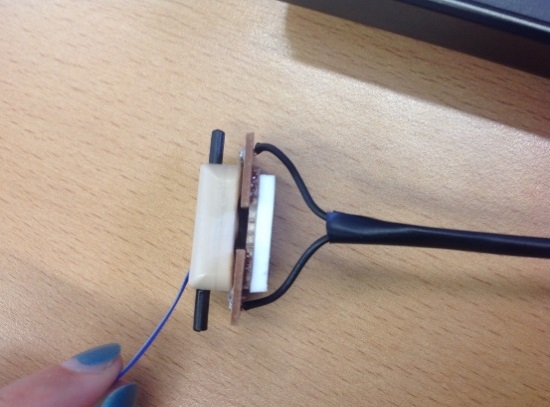

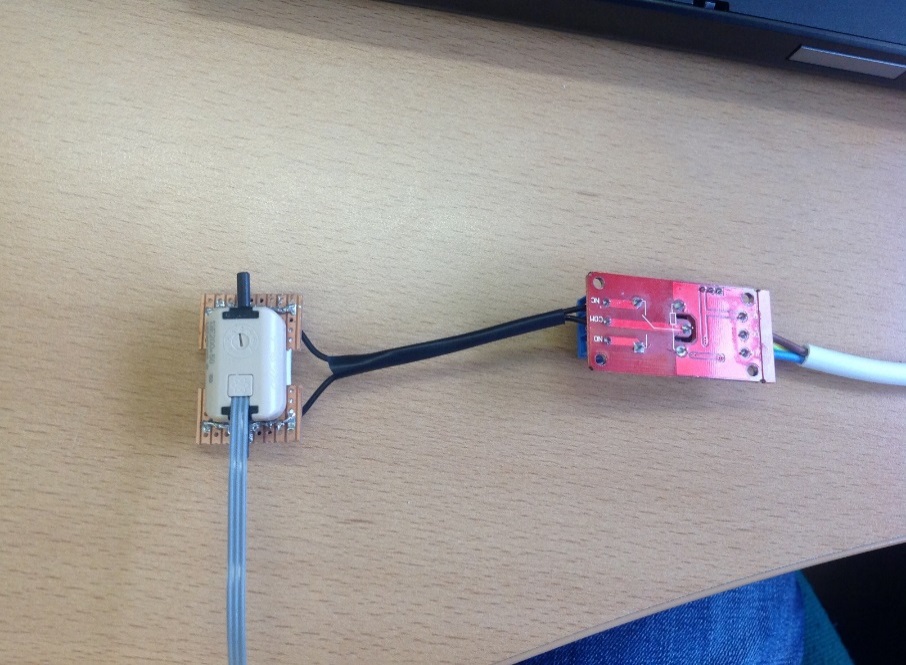


Switch (Normally Closed)

Insulating Foam

Wire conducting to each electrode

Stripboard ensures good contact with electrodes

**Figure 11.** Experimental setup for the assessment of delays

#### Relay switch operation

The switch is opened when current is passed through an electrical coil. **Figure 12** shows a 5V Songle switch with its outer casing removed. As the switch is activated the common terminal is pulled onto the coil, contacting the normally open part of the switch; when the current is removed the common terminal returns to contacting the normally closed terminal. The activation of the switch can be controlled by a signal sent from an Arduino to the input pin of the switch.


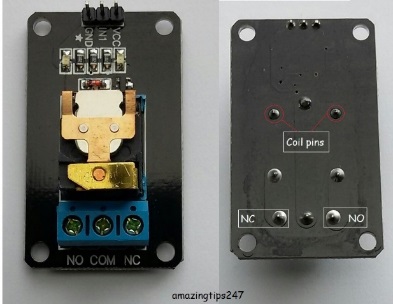

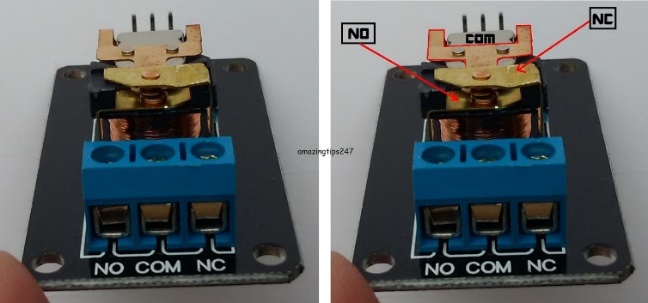

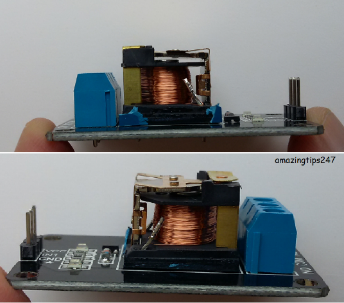

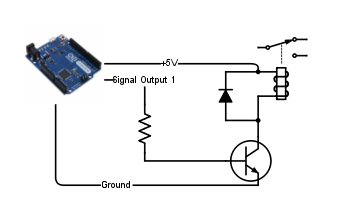


**Figure 12.** Internal view of the Songle SRD-05VDC-SL-C switch with circuit diagram taken from www.amazingtips247.co.uk article from 27/07/2015 entitled ‘Inside of a SRD-05VDC-SL-C relay and how to wire it up?’

### Checking the artificially generated signal level

To establish whether the voltage induced in the plates was of a suitable level to activate the prosthetic hand, a short test was undertaken.

The amplitude of a naturally generated myoelectric signal is different for each person, therefore, in clinical practice the myoelectrode gain settings are adjusted using the potentiometer on the rear of the myoelectrode, to produce a suitable post-processed signal to operate the prosthesis. To guide the clinician in selecting a suitable gain setting, clinicians typically use the Ottobock Myoboy® system to display the magnitude of the processed signal. To activate the prosthetic hand, a user should be able to comfortably achieve a processed myoelectric signal above a threshold, set within the Myoboy system by the manufacturers, at 24 (units undefined).

During the data collection for this thesis, as the researcher was not clinically qualified the gain settings on the users own prosthesis were not adjusted. Therefore, expert advice was sought as to the likely gain settings used in clinical practice, which were reported to lie between 3.5 and 5 for the majority of users.

To establish the amplitude of the signal that would be supplied to the hand by the experimental setup for electrodes configured with each gain setting, an electrode was connected to the Myoboy software (PAULA), allowing the post-processed signal level to be recorded. Due to the fact that some users may have their gain settings outside of the suggested 3.5-5 region, the whole range of available gain settings were evaluated. The gain was initially set at 1 and the electrode was activated using the circuit described above for a period of 5s, the switch was then closed for 5 seconds before re-opening a total of 4 times (**Figure 13**). This was repeated for each gain setting increasing in increments of 0.5 each time until the maximum gain setting of 7 was reached.


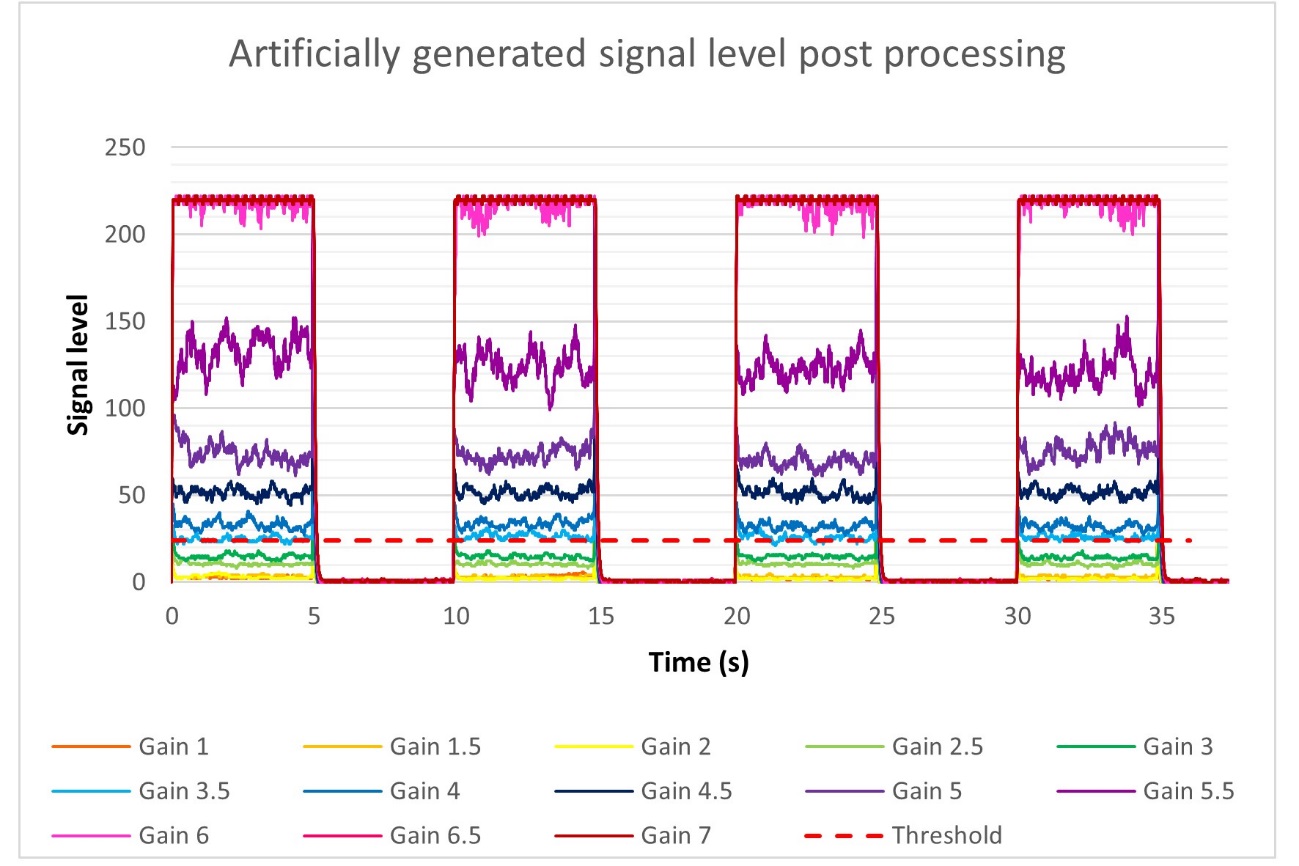


**Figure 13.** Output signal level recorded in Myoboy (PAULA) software for each electrode gain setting; when the gain setting reached 6, the signal exceeded the limit measurable using the Myoboy, explaining the clipping. The signal is sustained above the threshold (24) for gain settings >3.5.

**Figure 13** presents the resulting post-processed signal for each gain setting; the signal was sustained above the threshold level for gain settings >3.5 suggesting the experimental setup would be able to activate the prosthetic hand successfully for the majority if not all users.

Further testing was undertaken to establish exactly how the gain setting (and resulting signal amplitude) would impact on the measured delay (see below).

## Sensing the movement of the hand

Movement of the hand was measured using an electronic goniometer (Biometrics Ltd) attached across the proximal knuckle of the index finger (accuracy ± 2° measured over a range of ± 90°). A T9545 goniometer adaptor (Thought Technology Ltd accuracy ± 5%) and TT Sensor Isolator ST9405AM were used to return readings from the goniometer to an Arduino (referred to as “*Arduino 2*”) (see **Appendix 2 of Chadwell’s thesis** for more details <http://usir.salford.ac.uk/id/eprint/47264/>).

The angle data from the goniometer was relayed to Matlab for analysis via a serial interface. For each measurement the mean resting value (MRV) was calculated based on the first 80ms of data. A threshold of movement was set 1 degree above the MRV for hand opening, or 1 degree below the MRV for hand closing. The angle data was then double pass filtered using a 4^th^ order Butterworth filter with a cut off frequency of 20Hz. Onset of hand movement was taken as the moment the filtered angle data exceeded the threshold, and continued to increase by at least 5 degrees above the MRV (**Figure 14**).


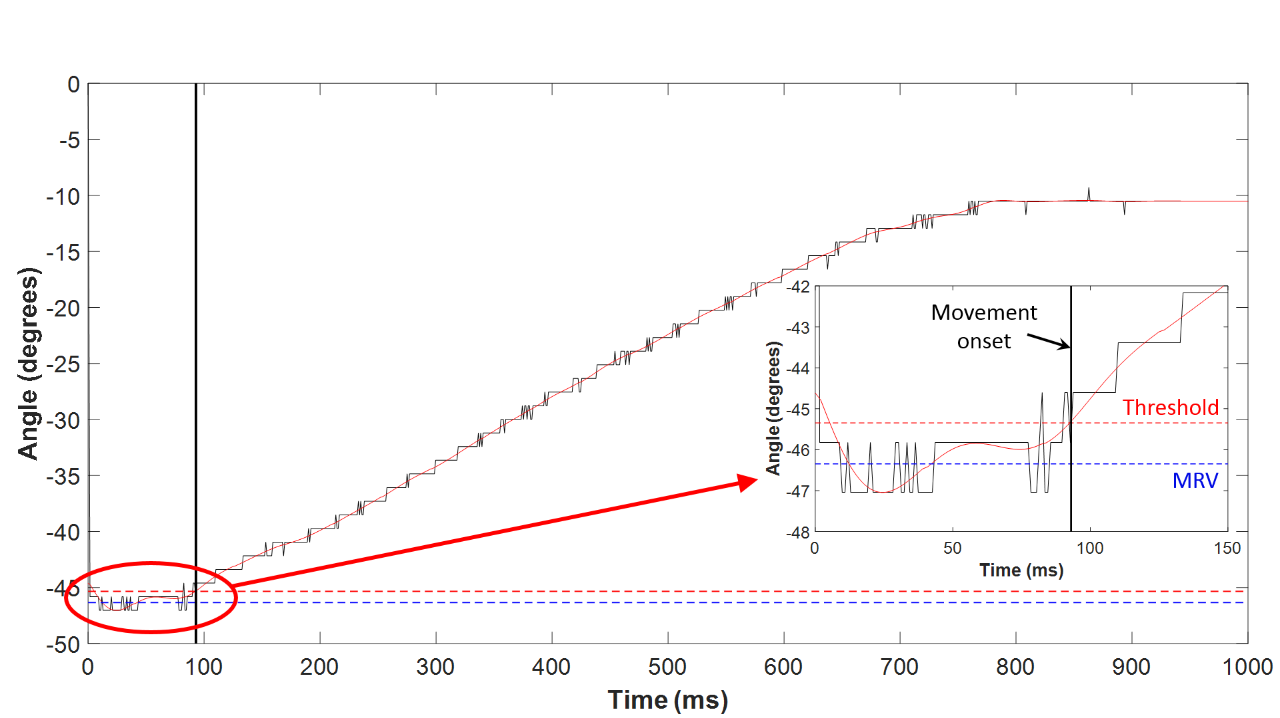


**Figure 14.** Example data (hand opening) showing the detection of hand movement onset.

## Checking for undesired delays introduced by the measurement equipment

In this section the potential delays introduced by each part of the measurement system are addressed, specifically:

- Is there a delay in the activation of the switch?
- Is there a delay in the voltage rise of the wires?
- Is there a delay in the time taken to initiate the goniometer recording?

### Measuring the delay in switch activation

To measure the delay in activation of the switch, a simple circuit was designed using the relay switch and a single Arduino (**Figure 15**). The normally closed poles of the relay switch were connected between two of the digital pins on the Arduino. One pin was configured as an output (“*Signal Output 2*”) and set to HIGH; the other was configured as an input pin (“*Signal Input 1*”). “*Signal Input 1*” was also connected to the Arduino’s ground pin via a resistor.


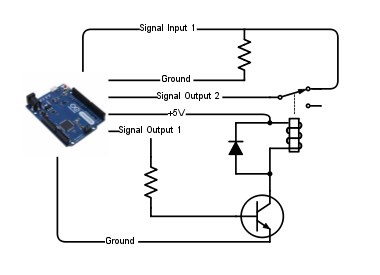


**Figure 15**. Diagram of circuit used to test the delay in the relay switch

A simple code was produced where the Arduino reported the clock time in microseconds before activating the relay switch and breaking the circuit. When the polarity of “*Signal Input 1*” was pulled from HIGH to LOW by the connection to ground (i.e. the switch had opened), the Arduino again reported the clock time in microseconds. The difference in these two timestamps represents the time taken to open the switch. This was repeated 50 times.

The mean switching delay measured over the 50 repeats was **2.675ms** (SD 0.032ms, min 2.612ms, max 2.776ms). This switching delay was highly consistent and could therefore be accurately accounted for in the overall delay measurement.

### Measuring the delay in stimulation of the wires

With the equipment available, it was not possible to measure the time taken between the switch opening, and the wires producing the appropriate voltage differential to activate the electrodes. The capacitance of a typical wire is minimal, and for the purposes of this study this delay was therefore assumed to be negligible.

### Measuring the delay in the goniometer

As noted above, goniometer data collection is controlled by “*Arduino 2*”. Using the equipment available, it was not possible to measure the delay between the movement of the goniometer and the movement data being received by the Arduino; furthermore, Biometrics do not report whether there is a significant delay between onset of movement and the outputting of voltages in their system. It can therefore be assumed that any delays are minimal and not worthy of reporting.

## Measuring the delay in prosthesis response

This section brings together the method of stimulating the electrodes and the method of detecting the onset of hand movement to calculate the electromechanical delay in the onset of hand movement (**Figure 10**).

Two Arduino Leonardo development boards (www.arduino.cc) were used to run the delay measurement setup. The setup was controlled via “*Arduino 1*”, whilst “*Arduino 2*” acted as an ADC for the goniometer (see **Appendix 2** **of Chadwell’s thesis** for more details <http://usir.salford.ac.uk/id/eprint/47264/>).

A digital pin on “*Arduino 1*” was configured as an output and was connected via a wire to the “*Recording Pin*” on “*Arduino 2*” (see **Appendix 2 of Chadwell’s thesis** for more details <http://usir.salford.ac.uk/id/eprint/47264/>). For each repeat of the delay measurement, “*Arduino 1*” first initiated data collection through “*Arduino 2*” and then immediately set the input pin of the switch high, opening the switch and stimulating the electrodes. After 1 second “*Arduino 1*” sent a signal to “*Arduino 2*” to stop the data recording, and then immediately set the input pin of the switch low, closing the switch and stopping the stimulation of the electrodes.

The angle data from the goniometer was imported from the recording buffer into Matlab for analysis. The delay was taken as the time from the onset of the goniometer data recording until the identified moment of hand movement onset, 2.675ms was then subtracted from this value to account for the delay in the switch activation.

## Establishing how the gain setting affects the delay

As noted above, when the gain of the myoelectrode is adjusted, the post-processed signal amplitude will change. To understand how this change in signal amplitude would affect the delay in the onset of hand movement, a short study was undertaken.

### Methodology

The hand was placed in a fully closed position and the electrode gain was set at the lowest setting (=1). The delay to the onset of hand opening (“*delay_O_C_*”) was measured 5 times. The gain was increased in increments of 0.5 up to the maximum gain setting of 7 and the “*delay_O_C_*” was measured 5 times at each of these gain settings.

The hand was then placed in a fully open position, and the delay to the onset of hand closing (“*delay_C_O_*”) was measured 5 times at each gain setting (1-7 in increments of 0.5).

This test was undertaken for each of: (1) a threshold controlled Steeper Select hand (owned by the research team), (2) a proportionally controlled Ottobock Myohand Variplus Speed (owned by the research team), and (3) the prosthesis owned by one of the pilot participants (Ottobock).

### Results

For the threshold controlled hand (Steeper Select) (**Figure 16**) the mean “*delay_O_C_*” to the onset of hand opening (from fully closed) was 91ms (SD = 17ms), whilst the mean “*delay_C_O_*” to the onset of hand closing (from fully open) was slightly shorter at 74ms (SD = 17ms). The gain setting did not appear to have an impact on the time taken for the hand to begin to move.

**Figure 16.** Delay recorded at each gain setting for a threshold controlled Steeper Select hand. Delays were measured from the extremes of hand open or closed.

**Figure 17** shows the results of the same test for a proportionally controlled hand (Ottobock Myohand Variplus Speed). Proportional control means that the motor torque is adjusted in relation to the amplitude of the myoelectric signal. At the lower gain settings (corresponding to lower motor torques for a given physiological EMG signal) the delay is significantly longer (delay at a gain of 1 = 474ms for opening or 203ms for closing). Once the torque exceeds a certain (unspecified) level the hand responds in a similar manner to the threshold controlled hand. It is worth noting that the “*delay_O_C_*” to the onset of hand opening (from a fully closed position) is approximately double the “*delay_C_O_*” to the onset of hand closing (from fully open). It is possible that this is caused by the time taken to achieve the required motor torque to overcome the resistance and backlash in the system. Additionally, in a fully closed position some deformation of the metal fingers occurs, the “*delay_O_C_*” may therefore be increased due to the time taken for the metal to return to its unloaded position before the fingers begin to open.

Finally **Figure 18** presents the results of the same test for the prosthetic hand used by one of the pilot prosthesis users. For this hand the gain setting does not appear to impact on the measured delays suggesting that the hand is configured to use threshold control. Similarly to the proportionally controlled hand, the “*delay_O_C_*” to the onset of hand opening (from a fully closed position) is significantly longer than the “*delay_C_O_*” to the onset of hand closing (from fully open). The mean delay to open the hand was 453ms (SD = 48ms) and to close it was only 91ms (SD = 15ms).

**Figure 17.** Delay recorded at each gain setting for a proportionally controlled Ottobock MyoHand VariPlus Speed. Delays were measured from the extremes of hand open or closed.

**Figure 18**. Delay recorded at each gain setting for a user owned prosthesis. Delays were measured from the extremes of hand open or closed.

### Conclusion

For a threshold controlled hand the gain setting does not appear to affect the delay in the time taken for the hand to start opening/closing; whereas, for a proportionally controlled hand, the delay to the onset of hand movement is longer at lower gain settings, until a plateau is reached.

It was also noted that for two of the three hands the time for the hand to begin opening from a fully closed position was significantly longer than the time taken for the hand to begin closing (from fully open). It was suggested that one of the primary reasons for this may be that when in a fully closed positon, the motor torque causes the metal finger/thumb to slightly deform. The “*delay_O_C_*” in the onset of hand opening may therefore be increased due to the additional time required for this deformation to relax before the finger/thumb begin to separate from each other.

## Establishing how the hand aperture affects the delay

In the previous section, the “*delay_O_C_*” to the onset of hand opening from a fully closed position (for the user’s prosthesis) was measured to be 453ms. In part of the early pilot work with the same prosthesis user the mean reaction time (made up of the user’s reaction time and the delay in prosthesis response) measured for the onset of hand opening was <300ms. The reaction time task was undertaken with the hand starting in a neutral position (neither open nor closed) suggesting that the hand starting aperture may have an impact on the delay to movement onset.

To allow a better understanding of the impact of the hand starting aperture on the measured delay, three short studies were undertaken.

1. Comparing the delay in the onset of hand opening from different neutral hand apertures (“*delay_O_N_*”)
2. Comparing the delay in the onset of hand opening from a neutral position (“*delay_O_N_*”) to the delay in the onset of hand opening from fully closed (“*delay_O_C_*”)
3. Comparing the delay in the onset of hand closing from a neutral position (“*delay_C_N_*”) to the delay in the onset of hand closing from fully open (“*delay_C_O_*”)

### Delay to open measured from different neutral apertures

This section addresses the “*delay_O_N_*” in the onset of hand opening, as measured with the hand starting at a number of different neutral (neither open nor closed) hand apertures. The following tests were undertaken for the proportionally controlled (Ottobock Variplus) hand, and for the user’s own prosthesis.

For each measurement the hand was placed in a neutral aperture (neither open nor closed) and the “*delay_O_N_*” to begin opening the hand was measured. The hand was then returned to a different neutral aperture, and the measurement was repeated; in total 20 measurements were undertaken, all at different starting apertures (excluding the extreme of fully closed). The full test (20 measurements) was undertaken at each gain setting (1-7 in increments of 0.5).

For the user’s own prosthesis, the results suggest that the initial (neutral position) hand aperture does not affect the delay in the time taken for the prosthetic hand to begin opening (for each gain setting p>0.05, and max Pearson’s R^2^ over all gain settings=0.17). **Figure 19** shows the results for 3 of the gain settings (1, 4, and 7).

**Figure 19**. Comparison of initial hand aperture against the delay to the onset of hand opening at a selection of gain settings for the user owned hand.

Similarly for the Ottobock Variplus hand, there was no clear correlation between the starting aperture and the “*delay_O_N_*” in the onset of hand opening (for each gain setting p>0.05, and max Pearson’s R^2^ over all gain settings=0.22). **Figure 20** presents the data for three of the gain settings (1, 4, 7). This figure supports the earlier findings that the delay is longer at the lower gain settings (gain = 1).

In summary, it was concluded that provided the hand was in a neutral starting position, the **specific aperture had no impact on the “*delay_O_N_*”** to the onset of hand opening.

**Figure 20.** Comparison of initial hand aperture against the delay to the onset of hand opening at a selection of gain settings for the Ottobock MyoHand VariPlus Speed.

### Delay to open measured from neutral aperture vs fully closed

Having established that there is no clear correlation between the hand aperture and the “*delay_O_N_*” in the onset of hand opening when starting in a neutral hand position, this section compares the “*delay_O_N_*” from a neutral position to the “*delay_O_C_*” from the extreme (fully closed).

Here the previously measured mean “*delays_O_N_*” to open the hand from a neutral position (from 20 measurements at each gain setting) are compared against the previously measured mean “*delays_O_C_*” to open the hand from a fully closed position (from 5 measurements at each gain setting).

In **Figure 21** the results for the user’s own prosthesis are displayed, whilst **Figure 22** displays the results for the Ottobock Variplus hand.

**Figure 21**. Delay to the onset of hand opening recorded at each gain setting for a user owned prosthesis. Delays were measured from the extreme of hand fully closed and from a range of neutral positions.

**Figure 22.** Delay to the onset of hand opening recorded at each gain setting for a proportionally controlled Ottobock MyoHand VariPlus Speed. Delays were measured from the extreme of hand fully closed and from a range of neutral positions.

For both hands, the delay in the onset of hand opening was significantly shorter when measured from a neutral aperture than when measured from a fully closed position. It was earlier demonstrated that the “*delay_O_C_*” was significantly longer than the “*delay_C_O_*”. These results show the length of the “*delay_O_N_*” to be similar to the previously measured “*delay_C_O_*”.

It was suggested previously that this additional delay to open the hand from a fully closed position may relate to the deformation of the metal fingers when the prosthesis is fully closed, and the backlash in the gears. These results support this theory.

### Delay to close measured from neutral aperture vs fully open

For the proportionally controlled Ottobock Variplus hand it was noted that at the lower gain settings, the “*delay_O_N_*” to open from a neutral position was in fact shorter than the “*delay_C_O_*” to close from a fully open position. The “*delay_C_N_*” to close from a neutral position was therefore also measured (20 times at each gain setting). The results were comparable to the hand opening from a neutral position (**Figure 23**).

**Figure 23.** Delay recorded at each gain setting for a proportionally controlled Ottobock MyoHand VariPlus Speed. Delays were measured from the extremes of hand open or closed. Both opening and closing were also measured from a range of neutral starting hand apertures.

From these results it is possible to conclude that the delay is significantly affected by hand posture (neutral vs the extremes). As in everyday life users may be opening their hands or closing their hands from a variety of different starting postures, it is clear that **the** **delay of any given prosthesis cannot be simply characterised by a single value**. In an attempt to address this, it was proposed that in the main study, delay values would be measured with the hand starting from both the extremes of aperture, and from a neutral hand aperture.

## Conclusions

These results of these short studies suggest that the measurement of delays is repeatable.

Due to the significant difference in the time to movement onset from the extremes of hand aperture when compared to the neutral positions, all four conditions will be assessed; this includes hand opening from fully closed and neutral, and hand closing from fully open and neutral. This may provide some useful information when assessing the onset delay for the functional task. During the main study it is proposed that the delay for each condition is measured five times.
