# Supplementary material for: Addressing unpredictability may be the key to improving performance with current clinically prescribed myoelectric prostheses
Source: Sci Rep. 2021 Feb 8;11:3300. doi: 10.1038/s41598-021-82764-6 (PMC7870859; doi:10.1038/s41598-021-82764-6)
Supplement: Supplementary file 3 — Supplementary Information 3. [file 41598_2021_82764_MOESM3_ESM.docx]

**Supplementary material to: Addressing unpredictability may be the key to improving performance with current clinically prescribed myoelectric prostheses**

**Gaze coding scheme inter-rater reliability study**

**Authors:** Chadwell A., Kenney L., Thies S., Head J., Galpin A., Baker R.

Coding scheme

Areas of interest

The task area was split into five areas of interest (AOI): (1) the prosthetic **hand**, (2) the Grasp Critical Area (**GCA**) of the cylinder, (3) the Location Critical Area (**LCA**) of the cylinder, (4) the **tube**, and (5) **other** (**Figure 24**).


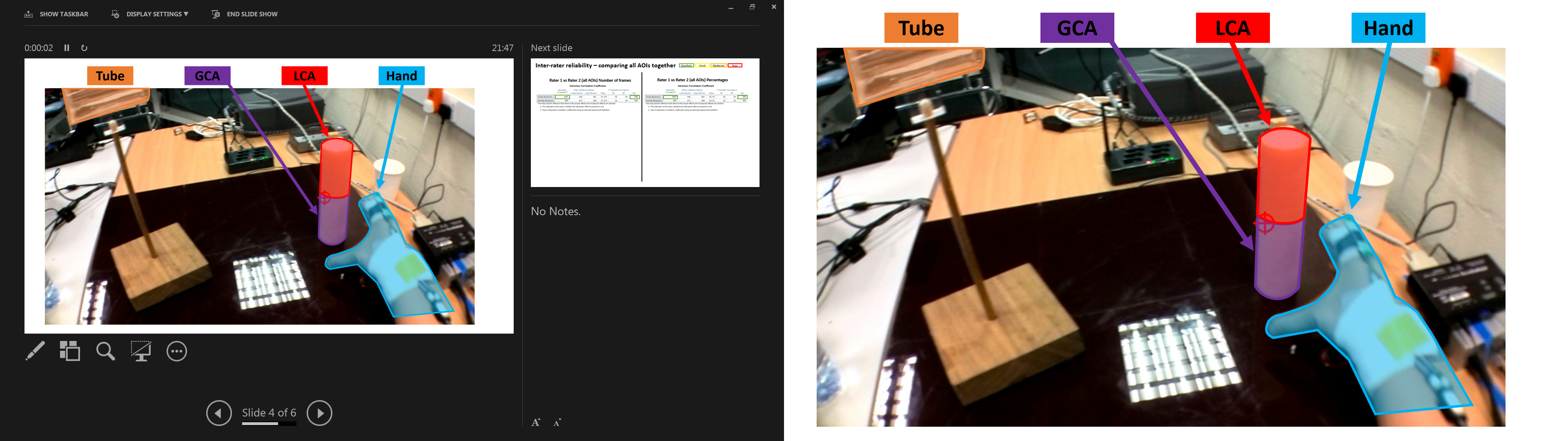


**Figure 24**. Areas of interest (AOIs) for cylinder task

Rules

The task was split into the “reach-to-grasp” phase, and the “transport” phase; the coding scheme was slightly different for each phase. Using the location of the centre of the crosshair, each frame of video data was coded according to these rules.

REACH-TO-GRASP

During “reach-to-grasp” the **LCA** and the **tube** were combined into a single AOI. Gaze at either of these areas corresponded to looking ahead to future portions of the task.

Coding options included: **Hand**, **GCA**, **LCA/Tube**, **Other** or **Missing data**.

The AOI **Hand** included any part of the prosthetic hand.

The **GCA** was defined as the bottom half of the cylinder, and the **LCA** was defined as the top half of the cylinder (**Figure 24**).

The **Tube** did not include the stand or the plastic block, however, if the crosshair was within 5mm of the opening of the tube (video size = 14.95*8.4cm) this was marked as **LCA/Tube**.

If the gaze was transitioning between positions, or the participant was looking at any other part of the task, this was coded as **Other**.

Data was marked as **Missing** **Data** if the participant blinked, or if the centre of the crosshair was outside of the field of view.

The following additional rules were put in place to cover periods during “reach-to-grasp” where the hand may move in front of the cylinder:

1. If the gaze is on the GCA and the hand moves into the area where the crosshair is this should continue to be coded as **GCA**, unless the pupils and the crosshair flick to a different location.
2. If the gaze is on the hand tracking its movement to the cylinder this should be coded as **Hand**, unless the pupils and the crosshair flick to a different location.
3. If the gaze is flicking between the hand and the cylinder, the coding should be consistent even if the hand and cylinder both fall under the crosshair. The rules above should be used as a guide.

TRANSPORT

During the “transport” phase the **LCA** and the **Tube** were combined into a single AOI. As the tube was transparent it was not possible to differentiate gaze at these two AOIs. The **Hand** and the **GCA** were also combined.

Coding options included: **Hand**/**GCA**, **LCA/Tube**, **Other** or **Missing data**.

The video data was two-dimensional, consequently when lifting and rotating the cylinder there were times when it was not clear which part of the cylinder the participant was looking at. The AOIs were therefore re-defined in a more restrictive manner. A line was drawn through the points where the index finger and thumb contact the cylinder (**Figure 25A**). The **LCA** included areas of the cylinder above this line, and the **GCA** included areas of the cylinder below this line (**Figure 25B**).

Furthermore, as the participants tracked the movement of the cylinder the crosshair would hover just off the edge of the cylinder. We therefore added a boundary region around the top of the cylinder and around the hand; if the crosshair hovered around the centre of the cylinder it was not clear which region they were looking at so this was left as **Other**. A line was drawn through the long axis of the cylinder (**Figure 25C**); perpendicular lines were then drawn which touched the top of the thumb, and the top edge of the cylinder (**Figure 25C**). If the crosshair was within 1cm of the top of the cylinder (video size = 14.95*8.4cm), and above the top line this was included in the **LCA** (**Figure 25D**). If the crosshair was within 1cm of the hand (video size = 14.95*8.4cm), and below the bottom line this was included in the **Hand/GCA** (**Figure 25D**).


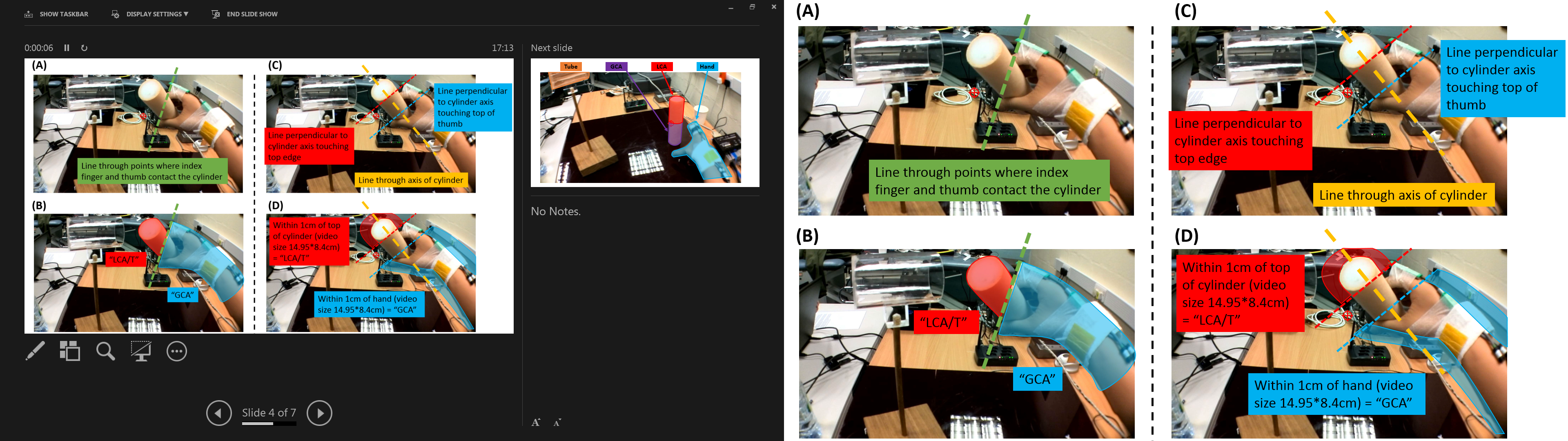


**Figure 25**. Re-defining the Areas of Interest (AOIs) for the “transport” phase. (A) and (B) relate to frames where the crosshair is on the hand or cylinder. (C) and (D) relate to frames where the crosshair is in the area surrounding the hand or cylinder.

The following rules were applied with respect to these boundary regions:

1. If the centre of the crosshair is within 5mm of the opening of the tube (video size = 14.95*8.4cm) code as **LCA/Tube**
2. If the centre of the crosshair is within 1cm of the top of the cylinder (defined according to a perpendicular line through the cylinder axis touching the edge of the cylinder – see **Figure 25C**) code as **LCA/Tube**
3. If the centre of the crosshair is within 1cm of the bottom of the cylinder/hand (defined according to a perpendicular line through the cylinder axis touching the top of the thumb– see **Figure 25C**) code as **Hand/GCA**
4. Once the cylinder begins to enter the tube rules 1 and 2 should be replaced by the following: If the centre of the crosshair is within 5mm of any part of the LCA or tube code as **LCA/Tube** (unless rule 3 is met)

If the gaze was transitioning between positions, or the participant was looking at any other part of the task, this was coded as **Other**.

Data was marked as **Missing** **Data** if the participant blinked, or if the centre of the crosshair was outside of the field of view.

Inter-rater reliability study

The data was independently coded by two separate people. The two raters agreed on a set of rules in advance. In total 6517 frames of data were coded by each rater. Statistical analysis of the coded data was undertaken using IBM SPSS statistics (v 24.0.0.1). The agreement between the raters was assessed using Cohen’s Kappa.

The results showed near perfect agreement between the two raters (κ = 0.909, p<.001). The gaze sequence from each of the two raters for each of the 20 assessed trials is presented **Figure 26,** and **Table 4** shows the crosstabulation of the agreement between the two raters output from SPSS. As can be seen in both **Figure 26** and **Table 4**, the main discrepancies between the two raters were between the **Hand** and the **GCA** during “reach-to-grasp”, and between the **LCA/Tube** and the **Hand/GCA** when releasing the cylinder at the end of the “transport” phase.

**Table 4.** Crosstabulation table for the agreement between the two raters

|  | | **Rater 2** | | | | | | |
| --- | --- | --- | --- | --- | --- | --- | --- | --- |
|  |  | **Hand** | **GCA** | **Hand/GCA** | **LCA/Tube** | **Missing Data** | **Other** | **Total** |
| **Rater 1** | **Hand** | 545 | 55 | 0 | 0 | 0 | 8 | 608 |
|  | **GCA** | 24 | 1021 | 0 | 17 | 0 | 2 | 1064 |
|  | **Hand/GCA** | 0 | 0 | 170 | 68 | 2 | 1 | 241 |
|  | **LCA/Tube** | 0 | 0 | 69 | 3686 | 6 | 7 | 3768 |
|  | **Missing Data** | 2 | 0 | 0 | 8 | 177 | 12 | 199 |
|  | **Other** | 7 | 23 | 0 | 53 | 1 | 553 | 637 |
|  | **Total** | 578 | 1099 | 239 | 3832 | 186 | 583 | 6517 |


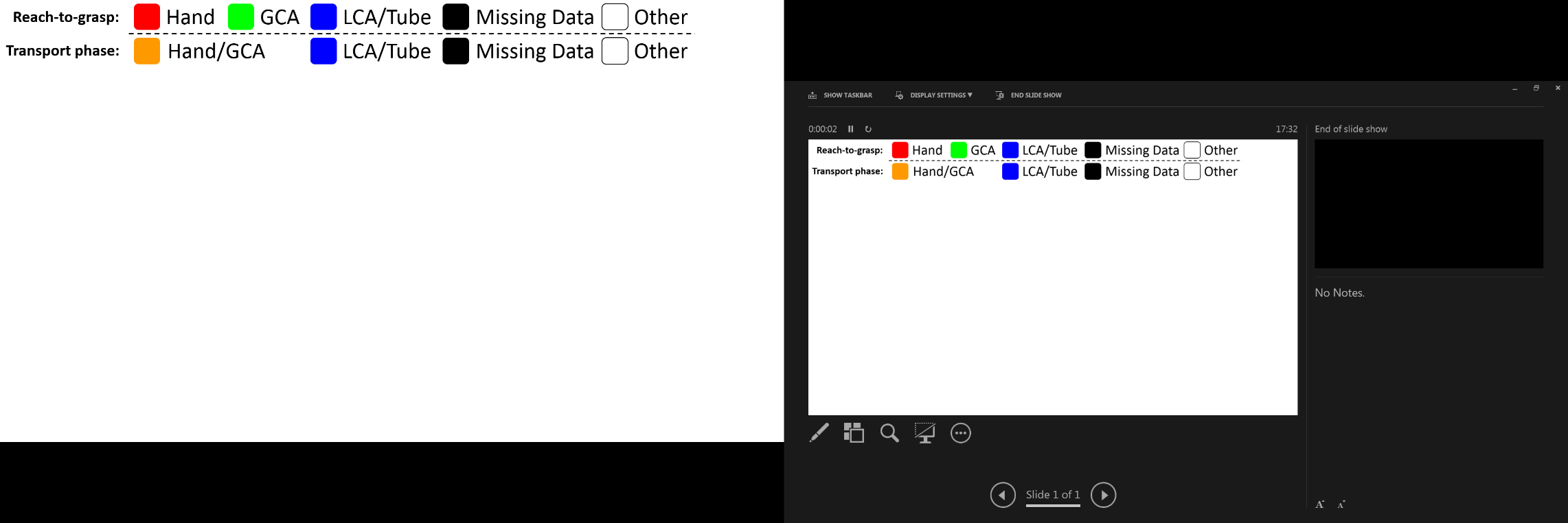

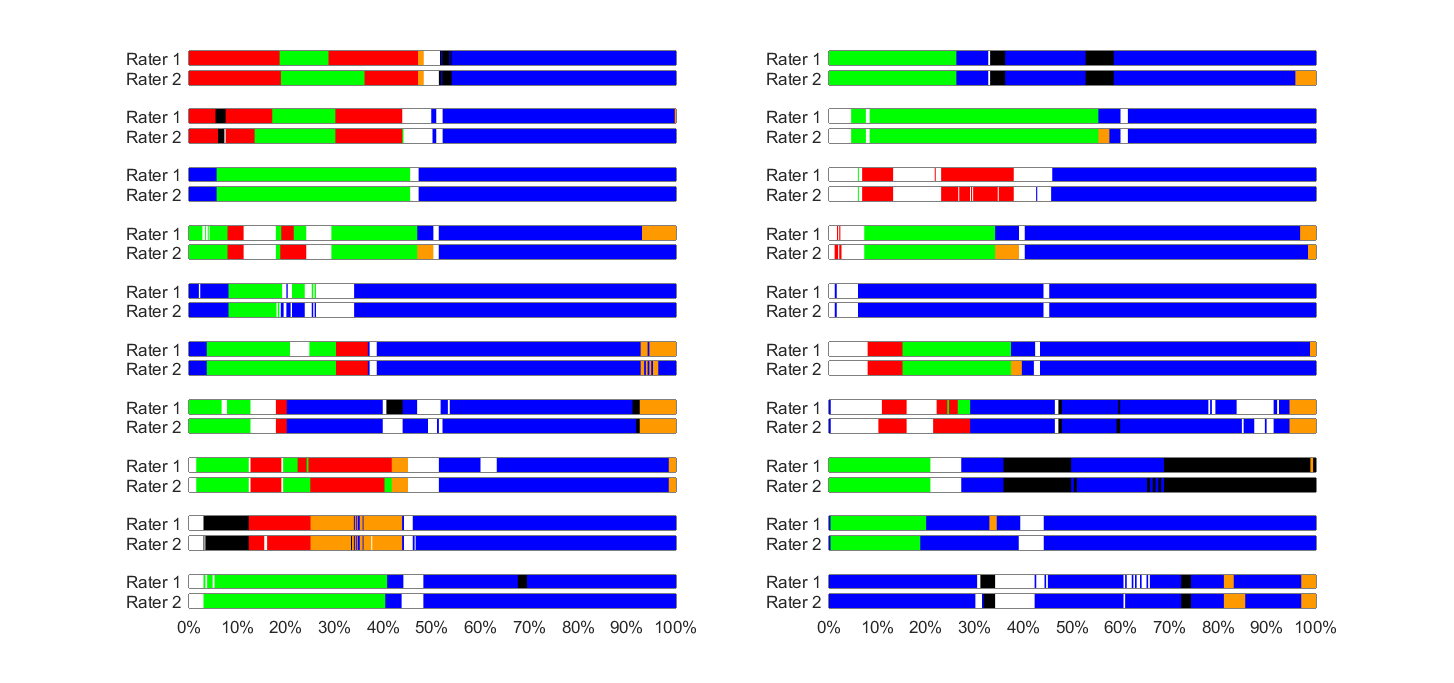


**Figure 26.** Coding plots for each trial to allow visual comparison of the gaze sequence between the two raters.
