# Supplementary material for: Addressing unpredictability may be the key to improving performance with current clinically prescribed myoelectric prostheses
Source: Sci Rep. 2021 Feb 8;11:3300. doi: 10.1038/s41598-021-82764-6 (PMC7870859; doi:10.1038/s41598-021-82764-6)
Supplement: Supplementary file 4 — Supplementary Information 4. [file 41598_2021_82764_MOESM4_ESM.docx]

**Supplementary material to: Addressing unpredictability may be the key to improving performance with current clinically prescribed myoelectric prostheses**

**Scatter plots showing correlations where τ_b_>0.3 or τ_b_<-0.3**

**Authors:** Chadwell A., Kenney L., Thies S., Head J., Galpin A., Baker R.

## EMG skill vs User Performance

**Significant correlations (p<0.05)**

**Correlations where p≥0.05**

**Figure 27.** Scatter plots showing the relationship between the measures representing the EMG skill of the user and their overall functionality and real-world use of the prosthesis where τ_b_>0.3 or <-0.3.

## Unpredictability vs User Performance

Assessment of desired activation

**Significant correlations (p<0.05)**

**Correlations where p≥0.05**

**Figure 28.** Scatter plots showing the relationship between the measures representing the desired activation of the prosthesis (reaction time spread and number of correct responses to reaction time tasks) and the prosthesis user’s overall functionality where τ_b_>0.3 or <-0.3.

Assessment of undesired activation

**Significant correlations (p<0.05)**

**Figure 29.** Scatter plots showing the relationship between the number of undesired activations of the prosthesis during the transitions (with an “ideal” skin-electrode interface) and the prosthesis user’s overall functionality where τ_b_>0.3 or <-0.3 and p<0.05.

**Correlations where p≥0.05**

**Figure 30.** Scatter plots showing the relationship between the number of undesired activations of the prosthesis during the transitions (with an “ideal” skin-electrode interface) and the prosthesis user’s overall functionality and real-world use of the prosthesis where τ_b_>0.3 or <-0.3 and p≥0.05.

**Significant correlations (p<0.05)**

**Figure 31.** Scatter plots showing the relationship between the number of undesired activations of the prosthesis during the transitions (using their own prosthesis and prosthetic socket) and the prosthesis user’s overall functionality and real-world use of the prosthesis where τ_b_>0.3 or <-0.3 and p<0.05.

**Correlations where p≥0.05**

**Figure 32.** Scatter plots showing the relationship between the number of undesired activations of the prosthesis during the transitions (using their own prosthesis and prosthetic socket) and the prosthesis user’s overall functionality where τ_b_>0.3 or <-0.3 and p≥0.05.

**Significant correlations (p<0.05)**

**Correlations where p≥0.05**

**Figure 33.** Scatter plots showing the relationship between the number of undesired activations of the prosthesis during the transitions (across all skin-electrode interface conditions) and the prosthesis user’s overall functionality where τ_b_>0.3 or <-0.3.

## Electromechanical Delays vs User Performance

**Significant correlations (p<0.05)**

**Figure 34.** Scatter plots showing the relationship between the electro-mechanical delay in the onset of hand movement for the prosthesis and the prosthesis user’s overall functionality and real-world use of the prosthesis where τ_b_>0.3 or <-0.3 and p<0.05.

**Correlations where p≥0.05**

**Figure 35.** Scatter plots showing the relationship between the electro-mechanical delay in the onset of hand movement for the prosthesis and the prosthesis user’s overall functionality and real-world use of the prosthesis where τ_b_>0.3 or <-0.3 and p≥0.05.
